# Allergic Rhinitis and Its Impact on Asthma (ARIA)‐EAACI Guidelines—2024–2025 Revision: Part I—Guidelines on Intranasal Treatments

**DOI:** 10.1111/all.70131

**Published:** 2025-12-01

**Authors:** Bernardo Sousa‐Pinto, Jean Bousquet, Rafael José Vieira, Holger J. Schünemann, Torsten Zuberbier, Antonio Bognanni, Alkis Togias, Boleslaw Samolinski, Arunas Valiulis, Sian Williams, Anna Bedbrook, Wienczyslawa Czarlewski, Maria Jose Torres, Mohamed H. Shamji, Mário Morais‐Almeida, G. Walter Canonica, Leticia de las Vecillas, Mark S. Dykewicz, Cristina Jacomelli, Ludger Klimek, Lucas Leemann, Olga Lourenço, Yuliia Palamarchuk, Nikolaos G. Papadopoulos, Ana Margarida Pereira, Marine Savouré, Sanna K. Toppila‐Salmi, Maria Teresa Ventura, Juan José Yepes‐Nuñez, Alvaro A. Cruz, Giorgio Ciprandi, Bilun Gemicioglu, Mattia Giovannini, Brigita Gradauskiene, Tuomas Jartti, Miloš Jeseňák, Piotr Kuna, Violeta Kvedariene, Désirée E. Larenas‐Linnemann, Amir HA Latiff, Yousser Mohammad, Ken Ohta, Padukudru A. Mahesh, Isabella Pali‐Schöll, Oliver Pfaar, Frederico S. Regateiro, Nicolas Roche, Luís Taborda‐Barata, Charlotte Suppli Ulrik, Marylin Valentin Rostan, Giovanni Viegi, Luo Zhang, Tari Haahtela, Ivan Cherrez‐Ojeda, Juan Carlos Ivancevich, Nikolai Khaltaev, Arzu Yorgancioglu, Baharudin Abdullah, Mona Al‐Ahmad, Maryam Ali Al‐Nesf, Rita Amaral, Julijana Asllani, Karl‐C Bergmann, Jonathan A. Bernstein, Michael S. Blaiss, Fulvio Braido, Paulo Camargos, Pedro Carreiro‐Martins, Thomas Casale, Lorenzo Cecchi, Alessandro Fiocchi, Antonio F. M. Giuliano, George Christoff, Ieva Cirule, Jaime Correia de Sousa, Elisio M. Costa, Stefano Del Giacco, Philippe Devillier, Dejan Dokic, Elham Hossny, Tomohisa Iinuma, Carla Irani, Zhanat Ispayeva, Kaja Julge, Igor Kaidashev, Kazi S. Bennoor, Helga Kraxner, Inger Kull, Marek Kulus, Maciej Kupczyk, Andriy Kurchenko, Stefania La Grutta, Neven Miculinic, Lan Le Thi Tuyet, Michael Makris, Branislava Milenkovic, Sang Min Lee, Stephen Montefort, Andre Moreira, Joaquim Mullol, Rachel Nadif, Alla Nakonechna, Hugo E. Neffen, Marek Niedoszytko, Dieudonné Nyembue, Robyn E. O'Hehir, Ismail Ogulur, Yoshitaka Okamoto, Heidi Olze, Oscar Palomares, Petr Panzner, Vincenzo Patella, Ruby Pawankar, Constantinos Pitsios, Todor A. Popov, Francesca Puggioni, Santiago Quirce, Agné Ramonaité, Marysia Recto, Maria Susana Repka‐Ramirez, Graham Roberts, Karla Robles‐Velasco, Menachem Rottem, Marianella Salapatas, Joaquin Sastre, Nicola Scichilone, Juan‐Carlos Sisul, Dirceu Solé, Manuel E. Soto‐Martinez, Milan Sova, Pongsakorn Tantilipikorn, Ana Todo‐Bom, Vladyslav Tsaryk, Ioanna Tsiligianni, Marilyn Urrutia‐Pereira, Erkka Valovirta, Tuula Vasankari, Dana Wallace, De Yun Wang, Margitta Worm, Osman M. Yusuf, Mihaela Zidarn, Sara Gil‐Mata, Manuel Marques‐Cruz, Bassam Mahboub, Ignacio J. Ansotegui, Antonino Romano, Werner Aberer, Maria Cristina Artesani, Elena Azzolini, Bruno Barreto, Joan Bartra, Sven Becker, Bianca Beghe, Attilio Boner, Ewa Borowiack, Jacques Bouchard, Melisande Bourgoin‐Heck, Luisa Brussino, Roland Buhl, José Antonio Castillo‐Vizuete, Denis Charpin, Niels H. Chavannes, Marta Chełmińska, Lei Cheng, Ekaterine Chkhartishvili, Seong H. Cho, Herberto Jose Chong‐Neto, Deepa Choudhury, Derek K. Chu, Cemal Cingi, Enrico Compalati, Raquel Albuquerque Costa, Olga Mariana Cunha, Biljana Cvetkovski, Victoria Cardona‐Dahl, Gennaro D'Amato, Janet Davies, Danilo Di Bona, Sandra N. Gonzalez Diaz, Maria V. Dimou, Maria Doulaptsi, Renato Ferreira‐da‐Silva, Radoslaw Gawlik, Mario Calvo‐Gil, Ozlem Goksel, Maximiliano R. Gómez, Maia Gotua, Christos Grigoreas, Ineta Grisle, Maria Antonieta Guzman, Rachel House Tan, Michael Hyland, Despo Ierodiakonou, Aspasia Karavelia, Paul Keith, Marta Kisiel, Tanja Soklic Kosak, Mitja Kosnik, Ilgim Vardaloglu Koyuncu, Vicky Kritikos, Justyna Litynska, Carlo Lombardi, Gilles Louis, Matteo Martini, Cem Meço, Eris Mesonjesi, Florin Mihaltan, Marcin Moniuszko, Robert N. Naclerio, Kari C. Nadeau, Sophia Neisinger, Markus Ollert, Michal Ordak, Giovanni Paoletti, Hae‐Sim Park, Elena Parmelli, Edgar Arturo Perdomo‐Flores, José Miguel Fuentes Pérez, Nhan Pham‐Thi, Emmanuel Prokopakis, Inês Ribeiro‐Vaz, Giovanni Rolla, Jan Romantowski, Philippe Rombaux, Philip W. Rouadi, Maia Rukhadze, Dermot Ryan, Ewelina Sadowska, Daiju Sakurai, Laila Salameh, Faradiba Serpa Sarquis, Elie Serrano, Jane Da Silva, Michael Soyka, Krzysztof Specjalski, Vesna Tomic‐Spiric, Katarina Stevanovic, Abirami Subramaniam, Maria Do Ceu Teixeira, Tuuli Thomander, Martina Vachova, Marianne van Hage, Pakit Vichyanond, Martin Wagenmann, Fanny Wai San Ko, Pascal Werminghaus, Paraskevi Vicky Xepapadaki, Yi‐Kui Xiang, Qintai Yang, Daniela Rivero Yeverino, Jaron Zuberbier, João A. Fonseca

**Affiliations:** ^1^ MEDCIDS—Department of Community Medicine, Information and Health Decision Sciences, Faculty of Medicine University of Porto Porto Portugal; ^2^ CINTESIS@RISE—Health Research Network, Faculty of Medicine University of Porto Porto Portugal; ^3^ Institute of Allergology Charité—Universitätsmedizin Berlin, Corporate Member of Freie Universität Berlin and Humboldt‐Universität Zu Berlin Berlin Germany; ^4^ Fraunhofer Institute for Translational Medicine and Pharmacology ITMP, Immunology and Allergology Berlin Germany; ^5^ ARIA (Allergic Rhinitis and Its Impact on Asthma) Montpellier France; ^6^ Department of Health Research Methods, Evidence, and Impact & Department of Medicine McMaster University Hamilton Ontario Canada; ^7^ Department of Biomedical Science Humanitas University, Pieve Emanuele Milan Italy; ^8^ Division of Allergy, Immunology, and Transplantation (DAIT) National Institute of Allergy and Infectious Diseases, NIH Bethesda Maryland USA; ^9^ Department of Prevention of Environmental Hazards, Allergology and Immunology Medical University of Warsaw Warsaw Poland; ^10^ Institute of Clinical Medicine and Institute of Health Sciences Medical Faculty of Vilnius University Vilnius Lithuania; ^11^ Clinic of Asthma, Allergy and Chronic Lung Diseases Vilnius Lithuania; ^12^ International Primary Care Respiratory Group IPCRG Edinburgh UK; ^13^ Medical Consulting Czarlewski Levallois France; ^14^ Allergy Unit Málaga Regional University Hospital of Málaga, Malaga University, ARADyAL Malaga Spain; ^15^ Immunomodulation and Tolerance Group and Allergy and Clinical Immunology Imperial College London London UK; ^16^ Allergy Center CUF Descobertas Hospital Lisbon Portugal; ^17^ Asthma & Allergy Unit IRCCS Humanitas Research Hospital, Rozzano Milan Italy; ^18^ Department of Allergy Hospital La Paz Institute for Health Research (IdiPAZ) Madrid Spain; ^19^ Section of Allergy and Immunology Saint Louis University School of Medicine Saint Louis Missouri USA; ^20^ “Respiriamo Insieme” Association, Asthma & Allergy Center Padova Italy; ^21^ Department of Otolaryngology, Head & Neck Surgery Universitätsmedizin Mainz Mainz Germany; ^22^ Center for Rhinology and Allergology Allergology & Rhinology Department Wiesbaden Germany; ^23^ Department of Political Science University of Zürich Zürich Switzerland; ^24^ RISE‐Health, Department of Medical Sciences Faculty of Health Sciences, University of Beira Interior Covilhã Portugal; ^25^ Finnish Meteorological Institute (FMI) Atmospheric Composition Research Helsinki Finland; ^26^ Allergy Department 2nd Pediatric Clinic, University of Athens Athens Greece; ^27^ PaCeIT—Patient Centered Innovation and Technologies, Center for Health Technology and Services Research (CINTESIS), Faculty of Medicine University of Porto Porto Portugal; ^28^ Allergy Unit Instituto and Hospital CUF Porto Portugal; ^29^ Barcelona Institute for Global Health (ISGlobal) Barcelona Spain; ^30^ Universitat Pompeu Fabra (UPF) Barcelona Spain; ^31^ CIBER Epidemiología y Salud Pública (CIBERESP) Barcelona Spain; ^32^ Department of Allergy, Skin and Allergy Hospital, Inflammation Center Helsinki University Hospital and University of Helsinki Helsinki Finland; ^33^ Department of Otorhinolaryngology University of Eastern Finland and the North Savo Wellbeing Services County Kuopio Finland; ^34^ University of Bari Medical School Bari Italy; ^35^ Institute of Sciences of Food Production National Research Council (ISPA‐CNR) Bari Italy; ^36^ School of Medicine Universidad de los Andes Bogotá D. C. Colombia; ^37^ Pulmonology Service, Internal Medicine Section Fundación Santa Fe de Bogotá, University Hospital Bogotá D. C. Colombia; ^38^ Fundacao ProAR and (Faculdade de Medicina da) Universidade Federal da Bahia Salvador Bahia Brazil; ^39^ Casa di Cura Villa Montallegro Allergology Department Genova Italy; ^40^ Department of Pulmonary Diseases and Institute of Pulmonology and Tuberculosis Istanbul University‐Cerrahpaşa, Cerrahpaşa Faculty of Medicine Istanbul Turkey; ^41^ Department of Health Sciences University of Florence Florence Italy; ^42^ Allergy Unit Meyer Children's Hospital IRCCS Florence Italy; ^43^ Department of Immunology and Allergology Lithuanian University of Health Sciences Kaunas Lithuania; ^44^ Department of Pediatrics and Adolescent Medicine University of Turku, Turku University Hospital Turku Finland; ^45^ Institute of Clinical Immunology and Medical Genetics, Department of Paediatrics and Adolescent Medicine, Department of Pulmonology and Phthisiology Jessenius Faculty of Medicine Martin Slovakia; ^46^ Comenius University Bratislava Slovakia; ^47^ University Teaching Hospital Martin Martin Slovakia; ^48^ Division of Internal Medicine, Asthma and Allergy Barlicki University Hospital, Medical University of Lodz Lodz Poland; ^49^ Institute of Clinical Medicine, Clinic of Chest Diseases and Allergology, Faculty of Medicine Vilnius University Vilnius Lithuania; ^50^ Institute of Biomedical Sciences, Department of Pathology, Faculty of Medicine Vilnius University Vilnius Lithuania; ^51^ Center of Excellence in Asthma and Allergy Médica Sur Clinical Foundation and Hospital México City Mexico; ^52^ Allergy & Immunology Centre Pantai Hospital Kuala Lumpur Kuala Lumpur Malaysia; ^53^ National Center for Research in Chronic Respiratory Diseases, Collaborating With WHO—EMRO Tishreen University School of Medicine Latakia Syria; ^54^ Al‐Sham Private University Pharmacy Department Damascus Syria; ^55^ Japan Antituberculosis Association (JATA) Fukujuji Hospital Tokyo Japan; ^56^ Department of Respiratory Medicine JSS Medical College, JSSAHER Mysuru Mysore Karnataka India; ^57^ Departement of Biological Sciences and Pathophysiology University of Veterinary Medicine Vienna Austria; ^58^ Section of Rhinology and Allergy, Department of Otorhinolaryngology, Head and Neck Surgery University Hospital Marburg, Philipps‐Universität Marburg Marburg Germany; ^59^ Allergy and Clinical Immunology Department Hospitais da Universidade de Coimbra, Unidade Local de Saúde de Coimbra Coimbra Portugal; ^60^ Center for Innovative Biomedicine and Biotechnology (CIBB), Faculty of Medicine University of Coimbra Coimbra Portugal; ^61^ Institute of Immunology, Faculty of Medicine University of Coimbra Coimbra Portugal; ^62^ UBIAir—Clinical & Experimental Lung Centre and CICS‐UBI Health Sciences Research Centre University of Beira Interior Covilhã Portugal; ^63^ Pneumologie AP‐HP Centre Université de Paris Cité, Hôpital Cochin Paris France; ^64^ UMR 1016 Institut Cochin Paris France; ^65^ Inserm Equipe D'epidémiologie Respiratoire Intégrative, CESP Villejuif France; ^66^ Department of Immunoallergology Cova da Beira University Hospital Centre Covilhã Portugal; ^67^ Department of Respiratory Medicine Copenhagen University Hospital‐Hvidovre Copenhagen Denmark; ^68^ Institute of Clinical Medicine University of Copenhagen Copenhagen Denmark; ^69^ Pediatrics, Allergy & Immunology, Latín American Society of Allergy Asthma & Immunology (SLAAi) Montevideo Uruguay; ^70^ Pulmonary Environmental Epidemiology Unit CNR Institute of Clinical Physiology Pisa Italy; ^71^ Department of Otolaryngology, Head and Neck Surgery Beijing TongRen Hospital and Beijing Institute of Otolaryngology Beijing China; ^72^ Skin and Allergy Hospital Helsinki University Hospital, and University of Helsinki Helsinki Finland; ^73^ Universidad Espíritu Santo Samborondón Ecuador; ^74^ Department of Allergology & Pulmonology Respiralab Research Group Guayaquil Guayas Ecuador; ^75^ Servicio de Alergia e Immunologia Clinica Santa Isabel Buenos Aires Argentina; ^76^ Global NCD Platform Geneva Switzerland; ^77^ Department of Pulmonary Diseases Celal Bayar University, Faculty of Medicine Manisa Turkey; ^78^ Department of Otorhinolaryngology—Head and Neck Surgery, School of Medical Sciences Universiti Sains Malaysia Kubang Kerian Kelantan Malaysia; ^79^ Microbiology Department College of Medicine, Kuwait University Kuwait City Kuwait; ^80^ Adult Allergy and Immunology Division—Hamad Medical Corporation Doha Qatar; ^81^ Department of Cardiovascular and Respiratory Sciences Porto Health School, Polytechnic Institute of Porto Porto Portugal; ^82^ Department of Women's and Children's Health, Paediatric Research Uppsala University Uppsala Sweden; ^83^ Department of Internal Medicine University of Medicine Tirana Albania; ^84^ Division of Immunology, Allergy and Rheumatology, Department of Medicine University of Cincinnati College of Medicine Cincinnati Ohio USA; ^85^ Department of Pediatrics Medical College of Georgia at Augusta University Augusta Georgia USA; ^86^ Department of Internal Medicine (DIMI) University of Genoa Genoa Italy; ^87^ Respiratory & Allergy Clinic IRCCS Ospedale Policlinico San Martino Genoa Italy; ^88^ Department of Pediatrics, Medical School Federal University of Minas Gerais Belo Horizonte Brazil; ^89^ NOVA Medical School/Comprehensive Health Research Centre (CHRC) Lisbon Portugal; ^90^ Serviço de Imunoalergologia Centro Hospitalar Universitário de Lisboa Central/ULS São José Lisbon Portugal; ^91^ Division of Allergy/Immunology University of South Florida Tampa Florida USA; ^92^ SOS Allergology and Clinical Immunology USL Toscana Centro Prato Italy; ^93^ Allergy, Bambino Gesù Children's Hospital Istituto di Ricovero e Cura a Carattere Scientifico (IRCCS) Rome Italy; ^94^ Department of Internal Medicine ‘A. Murri’ and Unit of Geriatric Immunoallergology University of Bari Medical School Bari Italy; ^95^ Faculty of Public Health Sofia Medical University Sofia Bulgaria; ^96^ Latvian Association of Allergists University Children Hospital Riga Latvia; ^97^ Life and Health Sciences Research Institute (ICVS), School of Medicine University of Minho Braga Portugal; ^98^ CINTESIS@RISE, Biochemistry Lab, Faculty of Pharmacy and Competence Center on Active and Healthy Ageing University of Porto Porto Portugal; ^99^ Department of Medical Sciences and Public Health and Unit of Allergy and Clinical Immunology University Hospital “Duilio Casula”, University of Cagliari Cagliari Italy; ^100^ VIM Suresnes, UMR 0892, Pôle Des Maladies Des Voies Respiratoires, Hôpital Foch Université Paris‐Saclay Suresnes France; ^101^ Medical Faculty Skopje University Clinic of Pulmology and Allergy Skopje Republic of North Macedonia; ^102^ Pediatric Allergy, Immunology and Rheumatology Unit Children's Hospital, Ain Shams University Cairo Egypt; ^103^ Department of Otorhinolaryngology Chiba University Chiba Japan; ^104^ Department of Internal Medicine and Infectious Diseases St Joseph University, Hotel Dieu de France Hospital Beirut Lebanon; ^105^ Department of Allergology and Clinical Immunology, Kazakhstan Association of Allergology and Clinical Immunology Kazakh National Medical University Almaty Kazakhstan; ^106^ Institute of Clinical Medicine Children's Clinic, Tartu University Tartu Estonia; ^107^ Poltava State Medical University Immunology & Allergology Department Poltava Ukraine; ^108^ Department of Respiratory Medicine National Institute of Diseases of the Chest and Hospital Dhaka Bangladesh; ^109^ Department of Otorhinolaryngology, Head and Neck Surgery Semmelweis University Budapest Hungary; ^110^ Department of Clinical Science and Education Södersjukhuset, Karolinska Institutet Stockholm Sweden; ^111^ Sach's Children and Youth Hospital, Södersjukhuset Stockholm Sweden; ^112^ Department of Pediatric Respiratory Diseases and Allergology Medical University of Warsaw Warsaw Poland; ^113^ Department of Clinical and Laboratory Immunology, Allergology and Medical Genetics Bogomolets National Medical University Kyiv Ukraine; ^114^ Institute of Translational Pharmacology (IFT)‐National Research Council (CNR) Palermo Italy; ^115^ Croatian Pulmonary Society Clinical Center for Pulmonary Diseases Zagreb Croatia; ^116^ Asthma, COPD Outpatient Care Unit University Medical Center Hô‐Chi‐Minh City Vietnam; ^117^ Allergy Unit “D Kalogeromitros”, 2nd Department of Dermatology and Venereology, National & Kapodistrian University of Athens “Attikon” University Hospital Athens Greece; ^118^ Clinic for Pulmonary Diseases, Clinical Center of Serbia, Faculty of Medicine University of Belgrade, Serbian Association for Asthma and COPD Belgrade Serbia; ^119^ Division of Respiratory Disease and Allergy, Department of Internal Medicine Dankook University College of Medicine Cheonan Republic of Korea; ^120^ Department of Medicine, Faculty of Medicine and Surgery University of Malta Msida Malta; ^121^ EPIUnit‐Institute of Public Health University of Porto, and Laboratory for Integrative and Translational Research in Population Health (ITR) Porto Portugal; ^122^ Serviço de Imunoalergologia Centro Hospitalar Universitário São João Porto Portugal; ^123^ Basic and Clinical Immunology Unit, Department of Pathology, Faculty of Medicine University of Porto Porto Portugal; ^124^ Rhinology Unit & Smell Clinic, ENT Department Hospital Clínic Barcelona Spain; ^125^ Clinical & Experimental Respiratory Immunoallergy, FRCB‐IDIBAPS, CIBERES University of Barcelona Barcelona Spain; ^126^ Université Paris‐Saclay UVSQ, Université Paris‐Sud Villejuif France; ^127^ Imperial College Healthcare NHS Trust London UK; ^128^ University of Liverpool Liverpool UK; ^129^ Center of Allergy, Immunology and Respiratory Diseases Santa Fe Argentina; ^130^ Department of Allergology Medical University of Gdansk Gdansk Poland; ^131^ ENT Department University Hospital of Kinshasa Kinshasa Democratic Republic of the Congo; ^132^ Allergy, Asthma and Clinical Immunology, Alfred Health and Department of Immunology, Central Clinical School Monash University Melbourne Victoria Australia; ^133^ Swiss Institute of Allergy and Asthma Research (SIAF) University of Zurich Davos Switzerland; ^134^ Chiba Rosai Hospital ENT Department Chiba Japan; ^135^ Chiba University Hospital Department of Otolaryngology, Head and Neck Surgery Chiba Japan; ^136^ Department of Otorhinolaryngology Charité—Universitätsmedizin Berlin Berlin Germany; ^137^ Department of Biochemistry and Molecular Biology, School of Chemistry Complutense University of Madrid Madrid Spain; ^138^ Department of Immunology and Allergology, Faculty of Medicine in Pilsen Charles University Prague Czech Republic; ^139^ Division of Allergy and Clinical Immunology Department of Medicine, “Santa Maria della Speranza” Hospital, Battipaglia Salerno Italy; ^140^ Agency of Health ASL Division of Allergy and Clinical Immunology, Department of Medicine Salerno Italy; ^141^ Postgraduate Programme in Allergy and Clinical Immunology University of Naples Federico II Naples Italy; ^142^ Department of Pediatrics Nippon Medical School Tokyo Japan; ^143^ Medical School University of Cyprus Nicosia Cyprus; ^144^ Clinic of Occupational Diseases University Hospital Sveti Ivan Rilski Sofia Bulgaria; ^145^ IRCCS Humanitas Research Center Personalized Medicine Asthma & Allergy, Rozzano Milan Italy; ^146^ Department of Pulmonology and Allergology Klaipeda National Hospital Klaipėda Lithuania; ^147^ Vilnius University Medical Faculty Vilnius Lithuania; ^148^ Division of Adult and Pediatric Allergy and Immunology University of the Philippines—Philippines General Hospital Manila Philippines; ^149^ Department of Allergy Clinics Hospital, National University San Lorenzo Paraguay; ^150^ Faculty of Medicine University of Southampton Southampton UK; ^151^ The David Hide Asthma and Allergy Centre St Mary's Hospital Newport Isle of Wight UK; ^152^ NIHR Southampton Biomedical Research Centre University Hospital Southampton NHS Foundation Trust Southampton UK; ^153^ LEADER Research Inc. Hamilton Ontario Canada; ^154^ Division of Allergy, Asthma and Clinical Immunology Emek Medical Center Afula Israel; ^155^ Rappaport Faculty of Medicine Technion‐Israel Institute of Technology Haifa Israel; ^156^ Aria Asthma, Rhinitis, Immunology & Allergy Department Athens Greece; ^157^ Allergy Service, Fundacion Jimenez Diaz Autonoma University of Madrid, CIBERES‐ISCIII Madrid Spain; ^158^ PROMISE Department University of Palermo Palermo Italy; ^159^ Allergy & Asthma Medical Director, CLINICA SISUL, FACAAI, SPAAI Asuncion Paraguay; ^160^ Division of Allergy, Clinical Immunology and Rheumatology, Department of Pediatrics Federal University of São Paulo São Paulo Brazil; ^161^ Division of Respiratory Medicine, Department of Pediatrics Hospital Nacional de Niños, Universidad de Costa Rica San Jose Costa Rica; ^162^ Department of Respiratory Medicine and Tuberculosis University Hospital Brno Czech Republic; ^163^ Department of Otolaryngology, Faculty of Medicine Siriraj Hospital Mahidol University Bangkok Thailand; ^164^ Imunoalergologia, Centro Hospitalar Universitário de Coimbra, Faculty of Medicine University of Coimbra Coimbra Portugal; ^165^ International Primary Care Respiratory Group IPCRG Aberdeen UK; ^166^ Health Planning Unit, Department of Social Medicine, Faculty of Medicine University of Crete Heraklion Greece; ^167^ Federal University of Pampa Department of Medicine Uruguaiana Brazil; ^168^ Department of Lung Diseases and Clinical Immunology University of Turku Turku Finland; ^169^ FiLHA Finnish Lung Health Association Helsinki Finland; ^170^ Department of Clinical Medicine, Pulmonary Diseases and Clinical Allergology University of Turku Turku Finland; ^171^ Nova Southeastern University College of Allopathic Medicine Fort Lauderdale Florida USA; ^172^ Department of Otolaryngology Yong Loo Lin School of Medicine, National University of Singapore Singapore Republic of Singapore; ^173^ Division of Allergy and Immunology, Department of Dermatology, Allergy and Venerology Charité Universitätsmedizin Berlin Berlin Germany; ^174^ The Allergy and Asthma Institute Allergy & Asthma Department Islamabad Pakistan; ^175^ University Clinic of Respiratory and Allergic Diseases Pulmonary & Allergy Department Golnik Slovenia; ^176^ Faculty of Medicine University of Ljubljana Ljubljana Slovenia; ^177^ Pulmonary Department Rashid Hospital, DUBAI Health Dubai UAE; ^178^ Research Institute of Medical and Health Sciences University of Sharjah Sharjah UAE; ^179^ Department of Allergy and Immunology Hospital Quironsalud Bizkaia Bilbao Spain; ^180^ Oasi Research Institute‐IRCCS Troina Italy; ^181^ BIOS S.P.A. Società Benefit Rome Italy; ^182^ Department of Dermatology Medical University of Graz Graz Austria; ^183^ Department of Allergy and Immunology Para State University Center—CESUPA Belém Brazil; ^184^ Allergy Department Hospital Clinic and IDIBAPS. Universitat de Barcelona Barcelona Spain; ^185^ RICORS de Enfermedades Inflamatorias (ISCIII) Madrid Spain; ^186^ Department for Otorhinolaryngology, Head and Neck Surgery University of Tübingen Tübingen Germany; ^187^ Respiratory Medicine University of Modena & Reggio Emilia, Azienda Ospedaliera‐Universitaria di Modena Modena Italy; ^188^ Pediatric Unit, Department of Surgical Sciences, Dentistry, Gynecology and Pediatrics University of Verona Verona Italy; ^189^ Evidence Prime Kraków Poland; ^190^ Evidence Prime Hamilton Ontario Canada; ^191^ Laval University Quebec City Quebec Canada; ^192^ Department of Pediatric Allergology Armand Trousseau University Hospital, Sorbonne University, AP‐HP Paris France; ^193^ French National Reference Center for Angioedema (CREAK) Saint‐Antoine University Hospital Paris France; ^194^ CRESS, Inserm, INRAE, HERA Team Paris Cité University Paris France; ^195^ Department of Medical Sciences University of Turin Turin Italy; ^196^ Allergy and Clinical Immunology Unit Mauriziano Hospital Torino Italy; ^197^ Department of Pulmonary Medicine Mainz University Hospital Mainz Germany; ^198^ Pneumology Department Hospital Universitari Dexeus Barcelona Spain; ^199^ CIBER of Respiratory Diseases, Group of Rhinitis, Rhinosinusitis and Nasal Polyps Area of Asthma, SEPAR Barcelona Spain; ^200^ Clinique Des Bronches, Allergie et Sommeil Hôpital Nord Marseille France; ^201^ Department of Public Health and Primary Care Leiden University Medical Centre Leiden the Netherlands; ^202^ National eHealth Living Lab Leiden the Netherlands; ^203^ Department of Pulmonology and Allergology Medical University of Gdańsk Gdańsk Poland; ^204^ Department of Allergology and Otorhinolaryngology The First Affiliated Hospital, Nanjing Medical University Nanjing China; ^205^ Allergist David Tvildiani Medical University Tbilisi Georgia; ^206^ University of South Florida College of Médicine, Division of Allergy‐Immunology, Department of Internal Medicine Tampa Florida USA; ^207^ Associate Professor of Pediatrics, Division of Allergy and Immunology Federal University of Parana Curitiba Brazil; ^208^ Paediatric Allergy Clinic, Department of Dermatology, Amersham Hospital NHS Hospital Trust Amersham UK; ^209^ Evidence in Allergy Group McMaster University and the Research Institute of St. Joe's Hamilton Hamilton Canada; ^210^ Eskisehir Osmangazi University Medical Faculty, ENT Department Eskisehir Turkey; ^211^ Scientific & Medical Department Lofarma S.p.A Milan Italy; ^212^ LAQV@REQUIMTE, Laboratory of Pharmacology, Department of Drug Sciences, Faculty of Pharmacy University of Porto Porto Portugal; ^213^ Woolcock Institute of Medical Research Sydney Australia; ^214^ Allergy Section, Department of Internal Medicine Hospital Vall D'hebron Barcelona Spain; ^215^ ARADyAL Research Network Barcelona Spain; ^216^ Division of Respiratory and Allergic Diseases, High Specialty Hospital ‘A Cardarelli’, and Respiratory Allergy School of Specialization in Respiratory Diseases Federico II University of Naples Naples Italy; ^217^ Centre for Immunology and Infection Control, School of Biomedical Sciences, Faculty of Health Queensland University of Technology Brisbane Australia; ^218^ Office of Research Metro North Hospital and Health Service Brisbane Australia; ^219^ University of Foggia Department of Medical and Surgical Science Foggia Italy; ^220^ Allergy and Clinical Immunology Centro Regional Hospital Universitario, Universidad Autónoma de Nuevo Leon Monterrey Mexico; ^221^ Department of Otorhinolaryngology, Head and Neck Surgery University of Crete, School of Medicine Heraklion Crete Greece; ^222^ Department of Internal Diseases, Allergology and Clinical Immunology Medical University of Silesia in Katowice Katowice Poland; ^223^ Pediatrics Department Universidad Austral de Chile Valvidia Chile; ^224^ Department of Pulmonary Medicine, Division of Immunology, Allergy and Asthma, Laboratory of Occupational and Environmental Respiratory Diseases, Faculty of Medicine Ege University, EgeSAM (Ege University Translational Pulmonary Research Center) Bornova Izmir Türkiye; ^225^ Faculty of Health Sciences Catholic University of Salta Salta Argentina; ^226^ Center of Allergy and Immunology, and Georgian Academy of Allergy Asthma, and Clinical Immunology Tbilisi Georgia; ^227^ Former President of the Hellenic Society of Allergology and Clinical Immunology, Department of Allergy and Clinical Immunology Air Force General Hospital Athens Greece; ^228^ Riga East University Hospital Riga Latvia; ^229^ Immunology and Allergy Division, Clinical Hospital University of Chile Santiago Chile; ^230^ Quality Use of Research Medicines Group Woolcock Institute of Medical Research Macquarie Park New South Wales Australia; ^231^ Macquarie University Macquarie Park New South Wales Australia; ^232^ Faculty of Health University of Plymouth Plymouth UK; ^233^ Department of Primary Care and Population Health University of Nicosia Medical School Nicosia Cyprus; ^234^ Otolaryngology Department General Hospital of Kalamata Kalamata City Greece; ^235^ Division of Clinical Immunology and Allergy, Department of Medicine McMaster University Hamilton Ontario Canada; ^236^ Occupational and Environmental Medicine, Department of Medical Sciences Uppsala University Uppsala Sweden; ^237^ ORL SOKLIC KOSAK Ljubljana Slovenia; ^238^ University Clinic of Respiratory and Allergic Diseases Golnik Slovenia; ^239^ Medical Faculty University of Ljubljana Ljubljana Slovenia; ^240^ Bagcilar Research and Training Hospital Department of Chest Diseases Istanbul Turkey; ^241^ Clinical Management Group, Woolcock Institute of Medical Research Macquarie University Sydney New South Wales Australia; ^242^ Sydney Pharmacy School, Faculty of Medicine and Health University of Sydney Sydney New South Wales Australia; ^243^ Departmental Unit of Allergology Clinical Immunology & Pneumology, Istituto Ospedaliero Fondazione Poliambulanza Brescia Italy; ^244^ Department of Public Health University of Liège Liège Belgium; ^245^ Department of Pneumology, GIGA I3 Research Group University of Liège Liège Belgium; ^246^ Allergy Unit, Department of Internal Medicine University Hospital AOU Delle Marche Ancona Italy; ^247^ Department of Clinical and Molecular Sciences Marche Polytechnic University Ancona Italy; ^248^ Department of Otorhinolaryngology, Head and Neck Surgery Ankara University Medical School Ankara Turkey; ^249^ Department of Otorhinolaryngology Head and Neck Surgery Salzburg Paracelsus Medical University Salzburg Austria; ^250^ Department of Otolaryngology, Head and Neck Surgery Cornell University, Weill Cornell Medical College New York New York USA; ^251^ Allergy & Clinical Immunology Department University Hospital Center “Mother Teresa” Tirana Albania; ^252^ UMF‐University of Medicine and Pharmacy ‘Carol Davila’, Pneumology Department National Institute of Pneumology ‘Marius Nasta’ Bucharest Romania; ^253^ Department of Allergology and Internal Medicine Medical University of Bialystok Bialystok Poland; ^254^ Department of Otolaryngology‐Head and Neck Surgery Johns Hopkins University Baltimore Maryland USA; ^255^ Department of Environmental Health, Harvard TH Chan School of Public Health and Department of Medicine, Division of Allergy and Inflammation Beth Israel Deaconess Hospital Boston Massachusetts USA; ^256^ Department of Infection and Immunity Luxembourg Institute of Health Esch‐sur‐Alzette Luxembourg; ^257^ Department of Dermatology and Allergy Centre Odense University Hospital, Odense Research Center for Anaphylaxis (ORCA) Odense Denmark; ^258^ Department of Pharmacotherapy and Pharmaceutical Care, Faculty of Pharmacy Medical University of Warsaw Warsaw Poland; ^259^ Department of Allergy and Clinical Immunology Ajou University School of Medicine Suwon Republic of Korea; ^260^ Clinical Epidemiology and Research Center, Department of Biomedical Sciences Humanitas University, Pieve Emanuele Milan Italy; ^261^ Otorhinolaryngology and Neck Surgery Unit San Juan de Dios National Hospital San Miguel El Salvador; ^262^ Allergist Private Practice Mexico City Mexico; ^263^ Ecole Polytechnique de Palaiseau Palaiseau France; ^264^ IRBA (Institut de Recherche Bio‐Médicale Des Armées) Bretigny sur Orge France; ^265^ Université Paris Cité Paris France; ^266^ Department of Otorhinolaryngology Cliniques Universitaires Saint‐Luc Brussels Belgium; ^267^ Department of Otolaryngology‐Head and Neck Surgery Eye and Ear University Hospital Beirut Lebanon; ^268^ Department of Otorhinolaryngology‐Head and Neck Surgery Dar Al Shifa Hospital Salmiya Kuwait; ^269^ Center Allergy & Immunology/Geomedi Teaching University Faculty of Medicine Tbilisi Georgia; ^270^ Usher Institute University of Edinburgh Edinburgh UK; ^271^ Department of Otorhinolaryngology Yamanashi University Yamanashi Japan; ^272^ Mohammed Bin Rashid University (MBRU) Dubai Health Dubai UAE; ^273^ Asthma Reference Center—School of Medicine of Santa Casa de Misericórdia of Vitória Espírito Santo Brazil; ^274^ Department of Otorhinolaryngology and Head & Neck Surgery CHU Rangueil‐Larrey Toulouse France; ^275^ Allergy Service University Hospital Professor Polydoro Ernani de São Thiago (HU‐UFSC/EBSERH) Florianopolis Brazil; ^276^ Department of Internal Medicine Federal University of Santa Catarina (UFSC) Florianopolis Brazil; ^277^ Otolaryngology‐HNS, University of Zurich University Hospital of Zurich Zurich Switzerland; ^278^ Clinic of Allergology and Immunology University Clinical Center of Serbia Belgrade Serbia; ^279^ Faculty of Medicine University of Belgrade Belgrade Serbia; ^280^ Department of Respiratory Medicine, Medical Professorial Unit Tallaght University Hospital & Trinity College Dublin Ireland; ^281^ Dr Agostinho Neto University Hospital Praia Cabo Verde; ^282^ Immunology Cabo Verde University, Faculty of Medicine Praia Cabo Verde; ^283^ The Heart and Lung Center Helsinki University Hospital and University of Helsinki Helsinki Finland; ^284^ Faculty of Medicine University of Porto Porto Portugal; ^285^ Department of Immunology and Allergology University Hospital and Faculty of Medicine in Pilsen Pilsen Czech Republic; ^286^ Charles University Prague Czech Republic; ^287^ Department of Medicine Solna, Division of Immunology and Respiratory Medicine Karolinska Institutet Stockholm Sweden; ^288^ Department of Clinical Immunology and Transfusion Medicine Karolinska University Hospital Stockholm Sweden; ^289^ Center for Molecular Medicine Karolinska University Hospital Stockholm Sweden; ^290^ Samitivej Allergy Institute Bangkok Thailand; ^291^ Division of Allergy and Immunology, Department of Pediatrics, Siriraj Hospital Mahidol University, Faculty of Medicine Bangkok Thailand; ^292^ Department of Otorhinolaryngology Heinrich Heine University Düsseldorf, Medical Faculty and University Hospital Düsseldorf Düsseldorf Germany; ^293^ The Chinese University of Hong Kong Department of Medicine and Therapeutics Hong Kong China; ^294^ ENT and Allergology Düsseldorf Germany; ^295^ Shanghai Skin Disease Hospital Tongji University School of Medicine Shanghai China; ^296^ The Third Affiliated Hospital of sun Yat‐Sen University Guangzhou China; ^297^ Servicio de Alergia e Inmunología clínica Hospital Universitario de Puebla Puebla México

**Keywords:** allergic rhinitis, guidelines, intranasal antihistamines, intranasal corticosteroids

## Abstract

**Background:**

Allergic rhinitis (AR) impacts quality of life, work and school productivity. Over the last years, an important body of evidence resulting from mHealth data has led to a better understanding of AR. Such advances have motivated an EAACI‐endorsed update of the Allergic Rhinitis and its Impact on Asthma (ARIA) guidelines (ARIA 2024–2025). This manuscript presents the ARIA 2024–2025 recommendations for intranasal treatments, one of the mainstays for AR management.

**Methods:**

The ARIA 2024–2025 guideline panel issued recommendations following the Grading of Recommendations, Assessment, Development, and Evaluation (GRADE) evidence‐to‐decision framework. Several sources of evidence were used to inform panel judgments and recommendations, including systematic reviews, evaluation of mHealth and pharmacovigilance data, as well as a survey of experts on costs.

**Results:**

Eleven guideline questions concerning intranasal treatments for AR were prioritized, leading to recommendations. Overall, these questions concern the choice between different classes of intranasal medications—most notably, intranasal corticosteroids (INCS), antihistamines (INAH), fixed combinations of INAH+INCS and decongestants—or between different individual medications within each class. Four questions had not been evaluated in previous ARIA guidelines, while for the other three there was a change in the strength or directionality of recommendations. Overall, recommendations point to the suggested use of INAH+INCS over INAH or INCS and INCS over INAH.

**Conclusion:**

This ARIA 2024–2025 article supports patients, their caregivers, and healthcare professionals in choosing an intranasal treatment. However, decisions on AR treatment should consider the clinical variability of the disease, patients' values, and the affordability of medications.

## Graphical abstract

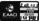

**Abbreviations**
ARIA: allergic rhinitis and its impact on asthma
GRADE: grading of recommendations, assessment, development, and evaluation
INAH: intranasal antihistamines
INCS: intranasal corticosteroids

## 1 Introduction

Allergic rhinitis (AR) is a common chronic disease [1, 2] that substantially impacts quality of life, work and school productivity, and social activities [3, 4, 5]. Several guidelines have been produced for AR management. In particular, the Allergic Rhinitis and its Impact on Asthma (ARIA) group first proposed its guidelines for AR and asthma multimorbidity in 2001 [6]. Subsequent revisions were published in 2008 [7], 2010 [8], 2016 [9], and 2020 [10], reflecting the development of new therapeutic options and/or improvements in its methodology. The ARIA 2010 and 2016 updates were developed following the Grading of Recommendations, Assessment, Development and Evaluation (GRADE) approach [9].

Further advances in the evidence landscape have motivated the development of a 2024–2025 revision of the ARIA guidelines (ARIA 2024–2025). In particular, over the last years, evidence from mHealth data has shed light on medication use patterns and adherence [11, 12], patients' satisfaction with treatments [13], and the impact of AR on work productivity [3].

Considering the above, ARIA 2024–2025 guidelines have been conceived as person‐centered, digitally enabled, and assisted by artificial intelligence (AI), using the GRADE approach [14]. This emphasis on a person‐centered guideline is relevant due to inter‐individual variability in (i) exposure and responses to triggers/allergens, (ii) impact of AR on daily life, (iii) values and preferences in relation to rhinitis health states, and (iv) disease management (ranging from self‐management to treatment by specialists).

This paper presents the first set of recommendations of ARIA 2024–2025, namely those on intranasal treatments for AR. Most guideline questions addressed in this paper concern intranasal corticosteroids (INCS), intranasal H_1_‐antihistamines (INAH) and fixed combinations of INAH+INCS. The target audience of these guidelines includes health professionals managing adults or children with AR, patients with AR, and health policymakers. ARIA 2024–2025 is supported by the European Academy of Allergy and Clinical Immunology (EAACI).

## 2 Questions Addressed by This Guideline

In ARIA 2024–2025, 42 questions on AR management were voted by guideline panel members as “prioritized questions” [15]. Among these, 11 concerned exclusively intranasal AR treatments and are addressed by this paper. The full set of questions is listed in Table 1, alongside their corresponding recommendations and capsule justifications.

**TABLE 1:** Recommendations of the ARIA 2024–2025 guidelines for the prioritized questions on intranasal treatments.

Recommendation	Capsule justification	Subgroup considerations	Implementation considerations
(A) New questions in ARIA 2024–2025			
**Should a combination of an INAH+INCS vs. no treatment be used for the treatment of AR?**			
In patients with AR in whom monotherapy is unlikely to lead to significant improvement in symptoms, we recommend using INAH+INCS over no treatment (strong recommendation|moderate CoE for seasonal AR and very low CoE for perennial AR)	INAH+INCS are effective in improving nasal symptoms, ocular symptoms and quality‐of‐life. INAH+INCS are overall safe, tendentiously cost‐effective and well accepted by patients	Recommendation applicable to children and adolescents	None specific
**Should any specific INCS vs. other INCS be used for the treatment of AR?**			
In patients with AR, we suggest using specific INCS (in particular, fluticasone furoate or fluticasone propionate) over others namely beclomethasone, budesonide, ciclesonide, mometasone and triamcinolone (conditional recommendation|low‐very low CoE)	Fluticasone furoate and fluticasone propionate are the most effective INCS in improving nasal symptoms and quality of life in patients with seasonal AR. Individual INCS display a similar safety and satisfaction profile. Costs vary across countries	In children and adolescents, evidence was not sufficient to support a specific recommendation	In low‐ and middle‐income countries, other INCS may be preferred based on local availability and affordability (budesonide is on the WHO List of Essential Medicines). From a planetary health perspective, locally produced generics may be preferrable.
**Should any specific combination of an INAH+INCS vs. other combination of an INAH+INCS be used for the treatment of AR?**			
In patients with AR, we suggest using azelastine‐fluticasone over olopatadine‐mometasone (conditional recommendation|moderate CoE)	Azelastine‐fluticasone is the most effective INAH+INCS in improving ocular symptoms and quality of life in patients with seasonal AR. Both INAH+INCS display a similar safety profile	In children and adolescents, we suggest either using azelastine‐fluticasone or olopatadine‐mometasone	In patients experiencing bitter taste with azelastine‐fluticasone, olopatadine‐mometasone may be preferred
**Should a combination of an INCS and an intranasal decongestant vs. an INCS alone be used for the treatment of AR?**			
In patients with AR, we suggest against using a combination of an INCS and an intranasal decongestant over an INCS alone (conditional recommendation|very low CoE)	Adding an intranasal decongestant to an INCS does not result in an increase in efficacy but is associated with a higher risk of adverse events, costs and impact on planetary health	Recommendation applicable to children and adolescents	In some specific situations, using intranasal decongestants for a short period of time when INCS are being introduced can be considered
(B) Questions with changed recommendation in terms of strength or directionality (compared to ARIA 2010/2016)			
**Should an INAH vs. no treatment be used for the treatment of AR?**			
In patients with AR, we recommend using INAH over no treatment (strong recommendation|moderate CoE)	INAH are effective in improving nasal symptoms, ocular symptoms and quality‐of‐life. INAH are overall safe, cost‐effective and moderately accepted by patients	Recommendation applicable to children and adolescents	None specific
**Should an intranasal decongestant vs. no treatment be used for the treatment of AR?**			
In adolescents and adults with AR, we suggest against using intranasal decongestants in the long term (longer than 5 days) over no treatment (conditional recommendation|very low CoE)	Decongestants display a trivial effect on nasal and ocular symptoms and are associated with an increased risk of adverse events	In pregnant women, children and the elderly, we suggest against using intranasal decongestants.	Recommendation concerning oxymetazoline, xylometazoline and tramazoline. The ARIA panel recommends against the use of ephedrine‐based decongestants
**Should a combination of an INAH+INCS vs. an INCS alone be used for the treatment of AR?**			
In patients with AR, we suggest using a combination of an INAH+INCS over an INCS alone (conditional recommendation|moderate CoE for seasonal AR and very low CoE for perennial AR)	INAH+INCS and INCS display a similar efficacy and safety profile. INAH+INCS are frequently cost‐effective and typically associated with a faster onset of action and higher satisfaction	Recommendation applicable to children and adolescents	INAH+INCS may be particularly favored in patients with more severe symptoms. In low‐income countries or low‐resource settings, INCS may be preferred
(C) Other questions in ARIA 2024–2025			
**Should an INCS vs. no treatment be used for the treatment of AR?**			
In patients with AR, we recommend using INCS over no treatment (strong recommendation|moderate CoE)	INCS are effective in improving nasal symptoms, ocular symptoms and quality‐of‐life. INCS are overall safe, cost‐effective and well‐accepted by patients	Recommendation applicable to children and adolescents	None specific
**Should an INCS vs. an INAH be used for the treatment of AR?**			
In patients with AR, we suggest using INCS over INAH (conditional recommendation|moderate CoE)	INCS and INAH display a similar efficacy and safety profile. INCS are typically less expensive (and/or cost‐effective) and associated with higher adherence and satisfaction	Recommendation applicable to children and adolescents	INCS may be particularly recommended for patients having taste alterations when using INAH. For patients with corticosteroid‐phobia, epistaxis or glaucoma, or having poor medication adherence, INAH may be considered
**Should a combination of an INAH+INCS vs. an INAH alone be used for the treatment of AR?**			
In patients with AR, we suggest using a combination of an INAH+INCS over an INAH alone (conditional recommendation|moderate CoE for seasonal AR and very low CoE for perennial AR)	INAH+INCS are more effective in improving nasal symptoms and quality of life. INAH+INCS display a similar safety profile. INAH+INCS tend to be cost‐effective and are associated with higher adherence and satisfaction	Recommendation applicable to children and adolescents, even though evidence is scarcer	None specific
**Should an INAH vs. an intranasal chromone be used for the treatment of AR?**			
In patients with AR, we suggest using INAH over intranasal chromones (conditional recommendation|very low CoE)	INAH appear to be more effective in improving nasal and ocular symptoms, and are associated with higher satisfaction. INAH and intranasal chromones display a similar safety profile	Recommendation applicable to children and adolescents	None specific

Abbreviations: AR, allergic rhinitis; CoE, certainty of evidence; INAH, intranasal antihistamines; INCS, intranasal corticosteroids.

## 3 Methodology

A full description of the methods used to develop recommendations in these guidelines is available elsewhere (Bousquet et al., under review). Here, we provide a brief methodological description to facilitate the interpretation of the guidelines.

### 3.1 Questions and Outcomes of Interest

In ARIA 2024–2025, four approaches were used for the development of guideline clinical questions, including (i) identification of questions answered by previous ARIA guidelines and US Practice Parameters, (ii) surveying of ARIA panel members (healthcare professional‐centered questions), (iii) identification of questions resulting from MASK‐air studies, and (iv) use of AI to support the generation of guideline questions [15] (patient‐centered questions). Questions were then subject to the GRADE formal process of prioritization [16] using GRADEpro [17].

Each question was assessed by considering the following set of outcomes (prioritized using GRADE formal processes [16]): nasal symptoms, ocular symptoms, quality of life, total symptoms, serious adverse events, and occurrence of any adverse event. Therefore, efficacy outcomes were nasal, ocular and total symptoms, as well as quality of life. Safety outcomes include any/total adverse events and serious adverse events.

### 3.2 Evidence Review and Development of Recommendations

For each question, we gathered evidence on the different criteria of the evidence‐to‐decision (EtD) framework, a systematic and transparent approach that aims to support the formulation of recommendations [18, 19]. The EtD comprises 12 criteria: priority, desirable and undesirable effects, certainty of evidence, values and preferences, balance of effects, resources required (and corresponding certainty of evidence), cost‐effectiveness, equity, acceptability, and feasibility. In addition, the ARIA 2024–2025 guidelines included a 13th criterion—planetary health [20, 21]—to account for the effects of interventions on both human health and the health of the planet.

Evidence on desirable and undesirable effects was obtained by conducting four systematic reviews (SRs) of randomized controlled trials (RCTs): (i) comparison of intranasal medications versus placebo in adults [22, 23, 24], (ii) comparisons among intranasal medications in adults [25, 26], (iii) comparisons among intranasal medications in children [27], and (iv) comparison of nonfixed treatment combinations in adults and children (in preparation). In addition, evidence on undesirable effects was complemented by an analysis of pharmacovigilance data. In particular, we queried VigiBase, the World Health Organization (WHO) global database of adverse event reports for medicines and vaccines [28].

For values and preferences, we conducted a SR of the literature [29]. For the remaining criteria, we performed nonsystematic evidence reviews, which were complemented by evidence from other sources. In particular, we have conducted a survey of ARIA experts assessing the availability and costs of different AR medications (in preparation). Furthermore, we analyzed MASK‐air direct patient data to obtain information on treatment acceptability (in particular, adherence, satisfaction, and use of co‐medication) and indirect costs associated with productivity losses. The WHO List of Essential Medicines was consulted to inform judgments on equity [30].

The voting members of the ARIA 2024–2025 panel (i.e., members without conflicts of interest) convened at recurrent online meetings (average of two meetings per PICO question, with 3–4 PICO questions being discussed per meeting), where they issued a judgment for each criterion of each question through GRADEpro PanelVoice [17]. Based on all the provided judgments, the panel issued a recommendation for the respective guideline question. Recommendations were worded following the GRADE working group guidance (see “How to use these guidelines” section) [31]. In addition, for each recommendation, we present (i) considerations for preschool and school‐aged children and adolescents (and, if evidence is available, other special populations, such as patients with asthma), and (ii) implementation considerations. The latter include, among others, aspects related to the application of recommendations in low‐ and middle‐income countries, or to concerns with specific adverse events.

For both judgments and recommendations, we sought consensus among voting members of the guideline panel. If consensus was not reached, a formal voting process was set. The final form of guideline recommendations and their wording, as well as the final guideline document, has been reviewed and approved by all panel members.

## 4 How to Use These Guidelines

The ARIA 2024–2025 guidelines are not intended to impose a standard of care for individual countries. They provide the basis for rational, informed decisions, so that their recommendations do not correspond to dictates. Recommendations provide guidance for typical patients but cannot account for all unique individual circumstances. Thus, clinicians are encouraged to tailor their practice considering the clinical presentation of each patient and the specificities of the respective local context, and to reach decisions via shared decision‐making.

For each question, in accordance with GRADE, we issued either a “strong” or “conditional” recommendation. The fact that a recommendation is “strong” or “conditional” reflects the panel's confidence that following it would result in a more beneficial outcome for patients and other interest‐holder categories (terminological clarification in Box 1). The wording of the recommendations reflects their strength, with “we recommend” implying a strong recommendation and “we suggest” implying a conditional recommendation. In each recommendation, we present information on the certainty of evidence across the different outcomes of interest (quality of the whole body of evidence, considering altogether desirable and undesirable effects; Box 1).

BOX 1 **Clarification of the terminology used in these guidelines.**

**Table:** 

Strength of recommendations Strong recommendation ○ *For patients*: Most patients in this situation would want the recommended course of action, and only a small proportion would not. ○ *For clinicians*: Most patients should receive the intervention. Adherence to a strong recommendation could be used as a quality criterion or performance indicator. Formal decision aids are not likely to be needed to help patients make decisions consistent with their values and preferences. ○ *For health care policy makers*: The recommendation can be adopted as a policy or performance measure in most situations. Conditional recommendation ○ *For patients*: Most patients in this situation would want the suggested course of action, but many would not. ○ *For clinicians*: Recognize that different choices will be appropriate for individual patients and that you must help each patient arrive at a management decision consistent with his or her values and preferences. Decision aids might be useful in helping patients to make decisions consistent with their values and preferences. ○ *For health care policy makers*: Policy making will require substantial debate and involvement of various stakeholders. Documentation of appropriate (e.g., shared) decision‐making processes can serve as a performance measure. Certainty of evidence: The certainty of evidence concerns how certain we are that the observed magnitude of desirable and undesirable anticipated effects lies on one side of a specified threshold or within a chosen range (reflecting the “quality” of available evidence). The certainty of evidence can be classified as “very low”, “low”, “moderate” or “high”. The certainty of evidence is independent of the directionality of the recommendation and of the effect sizes of the associations. Categorization of the effect sizes: The magnitude of the anticipated desirable and undesirable effects (“benefits and harms”) is classified by the GRADE working group as “trivial or none”, “small”, “moderate” or “large”. A trivial effect is observed when the magnitude of the effects is so small that it is not sufficiently important in terms of anticipated health consequences. Nontrivial effects can be considered “small”, “moderate” or “large” depending on the magnitude of effect sizes.

This manuscript provides a brief summary of the evidence underlying each recommendation (“brief justification”). Full EtDs for each question can be found online through links provided alongside each question.

Importantly, when summarizing our results on desirable and undesirable effects (“efficacy and safety”), we frequently report on the probability of differences between interventions being nontrivial (i.e., with corresponding effect sizes being sufficiently large that they are considered clinically important; Box 1 for terminological clarification).

## 5 Recommendations and Summary of Findings

Table 1 lists the recommendations for each prioritized question. Below, we discuss, for each question, the rationale underlying each recommendation; we first present questions that are new in relation to ARIA 2010/2016, followed by those for which the strength or directionality of recommendations has changed, and finally by the remainder. Of note, we do not specifically refer to patients' values and preferences in individual questions, as the same findings for values and preferences are applicable to all questions. In particular, we observed that patients generally (i) value the efficacy of interventions more over their safety, and (ii) consider nasal symptoms as those with the highest impact [29]. Table 2 presents, for each question, the judgment of the effect size and the certainty of the evidence for each outcome.

**TABLE 2:** Judgments on the effect sizes and certainty of evidence (CoE) assessments for each outcome in each prioritized question comparing each intervention to a comparator.

Question		Seasonal allergic rhinitis					Perennial allergic rhinitis				
		Nasal symptoms	Ocular symptoms	Quality of life	AE	Serious AE	Nasal symptoms	Ocular symptoms	Quality of life	AE	Serious AE
Should a combination of an INAH+INCS vs. no treatment be used for the treatment of AR?	Effect size	Moderate	Small	Moderate	Trivial	Trivial	Small	—^b^	Small	Trivial	Trivial
	CoE	Moderate	Moderate	Moderate	Moderate	Moderate	Very low	—^b^	Very low	Very low	Very low
Should any specific INCS vs. other INCS be used for the treatment of AR?	Effect size	Small	Trivial	Small	Trivial	Trivial	Trivial	Trivial	Small	Trivial	Trivial
	CoE	Very low/Low^a^	Very low/Low^a^	Very low/Low^a^	Very low/Low^a^	Very low/Low^a^	Very low/Low^a^	Very low	Very low/Low^a^	Very low/Low^a^	Very low/Low^a^
Should any specific combination of an INAH+INCS vs. other specific combination of an INAH+INCS be used for the treatment of AR?	Effect size	Trivial	Small	Small	Trivial	Trivial	—^b^	—^b^	—^b^	—^b^	—^b^
	CoE	Moderate	Moderate	Moderate	Moderate	Moderate	—^b^	—^b^	—^b^	—^b^	—^b^
Should a combination of an INCS and an intranasal decongestant vs. an INCS alone be used for the treatment of AR?	Effect size	Trivial	—^b^	Trivial	Small	Trivial	Trivial	—^b^	Trivial	Trivial	Trivial
	CoE	Very low	—^b^	Low	Very low	Very low	Very low	**—** ^b^	Very low	very low	Very low
Should an INAH vs. no treatment be used for the treatment of AR?	Effect size	Small	Small	Small	Trivial	Trivial	Small	—^b^	Small	Trivial	Trivial
	CoE	Moderate	High	High	Low	Low	High	—^b^	High	Moderate	Moderate
Should an intranasal decongestant vs. no treatment be used for the treatment of AR?	Effect size	Trivial	—^b^	Trivial	Trivial	—^b^	Trivial	—^b^	Trivial	Small	—^b^
	CoE	Very low	—^b^	Low	Moderate	—^b^	Low	—^b^	Very low	Low	—^b^
Should a combination of an INAH+INCS vs. an INCS alone be used for the treatment of AR?	Effect size	Trivial	Trivial	Trivial	Trivial	Trivial	Trivial	—^b^	Trivial	Trivial	Trivial
	CoE	Moderate	High	High	Moderate	Moderate	Very low	—^b^	Very low	Very low	Very low
Should an INCS vs. no treatment be used for the treatment of AR?	Effect size	Small	Small	Small	Trivial	Trivial	Small	Small	Small	Trivial	Trivial
	CoE	Moderate	High	Moderate	Moderate	Moderate	High	Moderate	Moderate	Moderate	Moderate
Should an INCS vs. an INAH be used for the treatment of AR?	Effect size	Trivial	Trivial	Trivial	Trivial	Trivial	Trivial	—^b^	Trivial	Trivial	Trivial
	CoE	Low	Moderate	High	Moderate	Moderate	High	—^b^	Low	Moderate	Moderate
Should a combination of an INAH+INCS vs. an INAH alone be used for the treatment of AR?	Effect size	Trivial	Trivial	Small	Trivial	Trivial	Small	—^b^	Trivial	Trivial	Trivial
	CoE	High	Moderate	High	Moderate	Moderate	Very low	—^b^	Very low	Moderate	Moderate
Should an INAH vs. an intranasal chromone be used for the treatment of AR?	Effect size	Small	Small	—^b^	Trivial	—^b^	—^b^	—^b^	—^b^	—^b^	—^b^
	CoE	Very low	Very low	—^b^	Very low	—^b^	—^b^	—^b^	—^b^	—^b^	—^b^

Abbreviations: AE, adverse events; AR, allergic rhinitis; INAH, intranasal antihistamines; INCS, intranasal corticosteroids. Colour code: Effect size: The darker the blue the larger the effect size. CoE: Green=High, Yellow=Moderate, Orange=Low, Red=Very low.

^a^ Most common CoE assessments for the considered comparisons.

^b^ No available evidence.

### 5.1 New Questions in ARIA 2024–2025

#### 5.1.1 Should a Combination of an Intranasal H_1_‐Antihistamine and an Intranasal Corticosteroid vs. no Treatment Be Used for the Treatment of AR?

**Link for the full EtD:** https://www.mask‐air.com/etd_nasal/01/.

**Context:** Fixed combinations of INAH+INCS are one of the mainstays for the treatment of AR, combining some of the advantages of INCS with those of INAH. However, INAH+INCS may not be affordable in all countries.

**Recommendation:** In patients with AR in whom monotherapy is unlikely to lead to significant improvement in symptoms, we recommend using INAH+INCS over no treatment. (Strong recommendation based on moderate certainty of evidence for seasonal AR and very low certainty of evidence for perennial AR).

- Considerations in children and adolescents: The recommendation is applicable to children and adolescents, with available studies having assessed children aged as low as 4 years old.
- Implementation considerations: None specific.

**Brief justification:** See online supplement.

#### 5.1.2 Should Any Specific Intranasal Corticosteroid vs. Another Intranasal Corticosteroid Be Used for the Treatment of AR?

**Link for the full EtD:** https://www.mask‐air.com/etd_nasal/02/.

**Context:** There are several INCS available, rendering it important not only to provide recommendations at a class level but also on what may be the most indicated individual INCS.

**Recommendation:** In adult patients with AR, we suggest using specific INCS (in particular, fluticasone furoate or fluticasone propionate) over others (namely, beclomethasone, budesonide, ciclesonide, mometasone and triamcinolone). (Conditional recommendation based on very low or low certainty of evidence for most comparisons).

- **Considerations in children and adolescents**: In children and adolescents, evidence was not sufficient to support recommending a specific INCS (insufficient number of primary studies). Daily doses in children aged < 12 years old may be lower (e.g., half) of those used in adults.
- **Implementation considerations**: In low‐ and middle‐income countries, other specific INCS may be preferred based on local availability and affordability (e.g., budesonide is on the WHO List of Essential Medicines). From a planetary health perspective, locally produced generics may be preferrable.

##### 5.1.2.1 Brief Justification

- Efficacy and safety:
  ○ A network meta‐analysis suggested that, in seasonal AR, fluticasone furoate and fluticasone propionate were the INCS displaying the highest probability of being more effective in improving nasal symptoms. In perennial AR, budesonide was the INCS displaying the highest probability of being the most effective in improving nasal symptoms, but it was only assessed by one trial.
  ○ In seasonal AR, beclomethasone and fluticasone furoate were the INCS having the highest probability of being the most effective in improving ocular symptoms. In perennial AR, fluticasone furoate was the most effective INCS for ocular symptoms.
  ○ In terms of rhinoconjunctivitis‐related quality of life (RQLQ), fluticasone furoate and fluticasone propionate were the INCS displaying the highest probability of being the most effective in seasonal AR. In perennial AR, the most effective INCS were fluticasone furoate and beclomethasone.
  ○ Similar frequencies and patterns of adverse events and serious adverse events were observed with the different INCS based on data from RCTs and pharmacovigilance.
- **Resources required, cost‐effectiveness and equity**: A survey of ARIA experts suggested that the least and most expensive INCS vary widely across countries. We did not identify any cost‐effectiveness study comparing INCS, but MASK‐air data suggests that mometasone and budesonide tend to be more frequently cost‐effective interventions compared to other INCS. Budesonide is the only INCS on the WHO List of Essential Medicines.
- **Acceptability and feasibility**: MASK‐air data suggest that fluticasone furoate and mometasone are the INCS associated with higher adherence. The different INCS are associated with similar levels of treatment satisfaction. However, fluticasone furoate and fluticasone propionate seem to be used more often in co‐medication than other individual INCS.
- **Planetary health**: No specific evidence was found in terms of comparative impact on planetary health.

#### 5.1.3 Should Any Specific Combination of an Intranasal H_1_‐Antihistamine and an Intranasal Corticosteroid vs. Another Combination of an Intranasal H_1_‐Antihistamine and an Intranasal Corticosteroid Be Used for the Treatment of AR?

**Link for the full EtD:** https://www.mask‐air.com/etd_nasal/03/.

**Context:** There are two widely used INAH+INCS—azelastine‐fluticasone and olopatadine‐mometasone, rendering it important not only to provide recommendations at a class level but also on what may be the most indicated individual INAH+INCS.

**Recommendation:** In adult patients with AR, we suggest using azelastine‐fluticasone over olopatadine‐mometasone. (Conditional recommendation based on moderate certainty of evidence for seasonal AR).

- **Considerations in children and adolescents**: In children and adolescents, we suggest either using azelastine‐fluticasone or olopatadine‐mometasone based on the available scarce evidence.
- **Implementation considerations**: In patients experiencing bitter taste with azelastine‐fluticasone, olopatadine‐mometasone may be preferred.

##### 5.1.3.1 Brief Justification

- **Efficacy and safety**:
  ○ A network meta‐analysis suggested that, compared to olopatadine‐mometasone, azelastine‐fluticasone is associated with a 23% probability of resulting in a nontrivial improvement in nasal symptoms in seasonal AR. For ocular symptoms and RQLQ, this probability was 56%.
  ○ For perennial AR, no evidence was available for the comparison between azelastine‐fluticasone versus olopatadine‐mometasone.
  ○ Similar frequencies of adverse events were observed with azelastine‐fluticasone and olopatadine‐mometasone (trivial difference). Serious adverse events associated with these interventions are rare and most of those reported in RCTs have been judged unlikely to be related to the treatment.
- **Resources required, cost‐effectiveness and equity**: A survey of ARIA experts suggested olopatadine‐mometasone to be more expensive than azelastine‐fluticasone in 8 out of 14 countries for which data were available. We did not identify any cost‐effectiveness study comparing these two medications. Neither azelastine‐fluticasone nor olopatadine‐mometasone are on the WHO List of Essential Medicines, but azelastine‐fluticasone seems to be available in a wider number of countries.
- **Acceptability and feasibility**: MASK‐air data suggest that azelastine‐fluticasone is associated with higher adherence and treatment satisfaction. However, azelastine‐fluticasone is associated with higher odds of being used in co‐medication (a proxy of poor rhinitis control) than olopatadine‐mometasone. In addition, there are studies on sensory attributes that favor olopatadine‐mometasone. Both azelastine‐fluticasone and olopatadine‐mometasone display a fast onset of action.

**Planetary health**: No specific evidence was found in terms of comparative impact on planetary health. Both branded products are manufactured in Asia, with olopatadine‐mometasone being distributed in a plastic vial and azelastine‐fluticasone being distributed in a glass vial.

#### 5.1.4 Should a Combination of an Intranasal Corticosteroid and an Intranasal Decongestant vs. an Intranasal Corticosteroid Alone Be Used for the Treatment of AR?

**Link for the full EtD**: https://www.mask‐air.com/etd_nasal/04/.

**Context**: Patients with AR using INCS often do co‐medication with intranasal decongestants (oxymetazoline, xylometazoline or tramazoline), particularly as the latter have a rapid onset and may help relieve nasal congestion.

**Recommendation**: In patients with AR, we suggest against using a combination of an INCS + intranasal decongestant over an INCS alone. (Conditional recommendation based on very low certainty of evidence).

- Considerations in children and adolescents: The recommendation is applicable to children and adolescents.
- Implementation considerations: This recommendation is particularly applicable to long‐term treatment (longer than 5 days). In some specific situations using intranasal decongestants for a short period of time—less than 5 days—when INCS are being introduced (to “compensate” for the slow onset of action of INCS) can be considered. However, if available and affordable, this can also be achieved with INAH+INCS (e.g., patients who cannot be treated for a long period of time with INAH+INCS due to costs or intolerance to bitter taste can have INAH+INCS for few days—to achieve fast symptom relief—followed by INCS). The ARIA panel recommends against the use of ephedrine‐based decongestants due to safety and legal concerns.

##### 5.1.4.1 Brief Justification

- Efficacy and safety
  ○ Primary studies assessing nasal symptoms were too different to allow for estimating meta‐analytical measures. However, these studies point to trivial differences when comparing the improvement of nasal symptoms between INCS + intranasal decongestants versus INCS alone.
  ○ For ocular symptoms, a single study indicated that “nonsignificant differences were identified for patients with seasonal AR” (no further information was provided).
  ○ Results from a network meta‐analysis suggested that INCS + intranasal decongestants and INCS are associated with a similar improvement in RQLQ, both in seasonal AR and perennial AR (trivial differences).
  ○ INCS + decongestants were associated with increased risk of adverse events compared to INCS (small but important difference). Of note, the long‐term use of decongestants has been linked to rhinitis medicamentosa. Serious adverse events associated with these interventions are rare and most of those reported in RCTs have been judged unlikely to be related to the treatment.
- **Resources required, cost‐effectiveness and equity**: A survey of ARIA experts suggested INCS + intranasal decongestants to represent up to more $200 per year than INCS alone for countries for which data were available. No cost‐effectiveness studies have been identified comparing INCS + intranasal decongestants versus INCS alone. There is both an INCS (budesonide) and a decongestant (xylometazoline) on the WHO List of Essential Medicines.
- **Acceptability and feasibility**: MASK‐air data suggest that INCS + decongestants are associated with lower adherence and treatment satisfaction. Intranasal decongestants display a faster onset of action than INCS.
- **Planetary health**: Considering that INCS + intranasal decongestants are not produced as fixed combinations, the additional use of intranasal decongestants implies the use of additional resources with environmental impact.

### 5.2 Questions With a Change in Recommendation Directionality and/or Strength in ARIA 2024–2025

#### 5.2.1 Should an Intranasal H_1_‐Antihistamine vs. no Treatment Be Used for the Treatment of AR?

**Link for the full EtD:** https://www.mask‐air.com/etd_nasal/05/.

**Context:** INAH are one possible therapeutic option in patients with AR, being often considered for patients with corticosteroid phobia and displaying a fast onset of action.

**Recommendation:** In patients with AR, we recommend using INAH over no treatment. (Strong recommendation based on moderate certainty of evidence).

- **Considerations in children and adolescents**: The recommendation is applicable to children and adolescents.
- **Implementation considerations**: None specific.

**Brief justification:** See online supplement.

#### 5.2.2 Should an Intranasal Decongestant vs. no Treatment Be Used For the Treatment of AR?

**Link for the full EtD:** https://www.mask‐air.com/etd_nasal/06/.

**Context:** Intranasal decongestants (oxymetazoline, xylometazoline and tramazoline) are frequently used by patients with AR, particularly considering that they are commonly sold over‐the‐counter and display a fast onset of action in nasal congestion.

**Recommendation:** In patients with AR, we suggest against using intranasal decongestants in the long term (longer than 5 days) over no treatment. (Conditional recommendation based on very low certainty of evidence).

- Considerations in specific age groups and conditions: For preschool and young school‐aged children (< 12 years), there should be avoidance of intranasal decongestants. There should also be avoidance of intranasal decongestants in pregnant women, especially in the first trimester, considering the potential teratogenic effects of nasal decongestants. Considering the risk of serious adverse events, the use of intranasal decongestants in the elderly is also discouraged.
- Implementation considerations: This recommendation concerns oxymetazoline, xylometazoline and tramazoline. The panel suggests that the use of intranasal decongestants should be restricted to short‐term relief (not longer than 5 days and preferably shorter) of nasal congestion. The ARIA panel also recommends against the use of ephedrine‐based decongestants due to safety and legal concerns.

**Brief justification:** See online supplement.

#### 5.2.3 Should a Combination of an Intranasal H_1_‐Antihistamine and an Intranasal Corticosteroid vs. an Intranasal Corticosteroid Alone Be Used for the Treatment of AR?

**Link for the full EtD:** https://www.mask‐air.com/etd_nasal/07/.

**Context:** INCS+INAH offer some advantages in relation to INCS in terms of onset of action and, potentially, effectiveness. However, INCS are more widely available and are more affordable.

**Recommendation:** In patients with AR, we suggest using a fixed combination of an INAH and INCS over an INCS alone. (Conditional recommendation based on moderate certainty of evidence for seasonal AR and on very low certainty of evidence for perennial AR).

- **Considerations in children and adolescents**: The recommendation is applicable to children and adolescents.
- **Implementation considerations**: Aspects such as adherence, baseline severity and history of medication use may be relevant to be considered. Fixed combinations of INAH+INCS may be particularly favored in patients with severe symptoms. In low‐income countries or low‐resource settings, INCS may be preferred.

##### 5.2.3.1 Brief Justification

- Efficacy and safety
  ○ A network meta‐analysis suggested that INAH+INCS and INCS are associated with a similar improvement in nasal and ocular symptoms, as well as in RQLQ, both in seasonal AR and perennial AR (trivial differences).
  ○ Similar frequencies of adverse events were observed with INAH+INCS and INCS (trivial difference). Serious adverse events associated with these interventions are rare and most of those reported in RCTs have been judged unlikely to be related to the treatment.
- **Resources required, cost‐effectiveness and equity**: A survey of ARIA experts suggested INAH+INCS to be more expensive than INCS in 34 out of 36 countries for which data were available. However, INAH+INCS are likely cost‐effective in most countries both when considering a willingness‐to‐pay of $50,000/QALY gained or of one time the GDP per capita/QALY gained (the only exception may be some low‐ and middle‐income countries). One INCS—budesonide—is on the WHO List of Essential Medicines, but the same does not occur with INAH+INCS.
- **Acceptability and feasibility**: MASK‐air data suggest that INAH+INCS are associated with higher treatment satisfaction and with lower odds of being used in co‐medication (a proxy of poor rhinitis control). In addition, INAH+INCS display a faster onset of action.

**Planetary health**: No specific evidence was found in terms of comparative impact on planetary health, even though it is possible that the production of two active compounds may have a higher environmental impact than the production of one.

### 5.3 Other Questions Evaluated in ARIA 2024–2025

#### 5.3.1 Should an Intranasal Corticosteroid vs. no Treatment Be Used for the Treatment of AR?

**Link for the full EtD:** https://www.mask‐air.com/etd_nasal/08/.

**Context:** INCS are one of the mainstays of the treatment of AR, being widely available. Budesonide is listed in the WHO List of Essential Medicines.

**Recommendation:** In patients with AR, we recommend using INCS over no treatment. (Strong recommendation based on moderate certainty of evidence).

- **Considerations in children and adolescents**: The recommendation is applicable to children and adolescents.
- **Implementation considerations**: None specific.

**Brief justification:** See online supplement.

#### 5.3.2 Should an Intranasal Corticosteroid vs. an Intranasal H_1_‐Antihistamine Be Used for the Treatment of AR?

**Link for the full EtD:** https://www.mask‐air.com/etd_nasal/09/.

**Context:** INCS and INAH are two of the most commonly used classes for the treatment of AR. These two treatment classes have distinct profiles in terms of acceptability and affordability, among others.

**Recommendation:** In patients with AR, we suggest using INCS over INAH. (Conditional recommendation based on moderate certainty of evidence).

- Considerations in children and adolescents: The recommendation is applicable to children and adolescents.
- Implementation considerations: INCS may be particularly recommended for patients having taste alterations when using INAH. For patients with corticosteroid‐phobia, epistaxis secondary to INCS or glaucoma, or having poor medication adherence, INAH may be the preferred intervention.

##### 5.3.2.1 Brief Justification

- Efficacy and safety
  ○ A network meta‐analysis suggested that, compared to INAH, INCS are associated with a trivial improvement in nasal symptoms and in RQLQ in seasonal and perennial AR.
  ○ For ocular symptoms, either differences between treatments were trivial (seasonal AR) or evidence was not found (perennial AR).
  ○ Similar frequencies of adverse events were observed with INCS and INAH (trivial difference). Serious adverse events associated with these interventions are rare and most of those reported in RCTs have been judged unlikely to be related to the treatment. Although rare, pharmacovigilance data suggested glaucoma to be more frequent with INCS than INAH.
- **Resources required, cost‐effectiveness and equity**: A survey of ARIA experts suggested INAH to be more expensive than INCS in 21 out of 29 countries for which data were available. In addition, in most countries where INCS are more expensive than INAH, INCS were found to be cost‐effective when considering a willingness‐to‐pay of $50,000/Quality Adjusted Life Years (QALY) gained or of one time the Gross Domestic Product (GDP) per capita/QALY gained. One INCS—budesonide—is on the WHO List of Essential Medicines, but the same does not occur with INAH. In addition, INCS are available in more countries than INAH.
- **Acceptability and feasibility**: MASK‐air data suggest that INCS are associated with higher adherence and treatment satisfaction, as well as with lower odds of being used in co‐medication (a proxy of poor rhinitis control). However, INAH display a faster onset of action.
- **Planetary health**: No specific evidence was found in terms of comparative impact on planetary health.

#### 5.3.3 Should a Combination of an Intranasal H_1_‐Antihistamine and an Intranasal Corticosteroid vs. an Intranasal H_1_‐Antihistamine Alone Be Used for the Treatment of AR?

**Link for the full EtD:** https://www.mask‐air.com/etd_nasal/10/.

**Context:** INCS+INAH have been proposed as the first‐line treatment of AR by previous guidelines. However, some patients are corticosteroid‐phobic or have glaucoma. Thus, INAH alone may be of interest.

**Recommendation:** In patients with AR, we suggest using a fixed combination of an INAH+INCS over an INAH alone. (Conditional recommendation based on moderate certainty of evidence for seasonal AR and on very low certainty of evidence for perennial AR).

- **Considerations in children and adolescents**: The recommendation is applicable to children and adolescents, even though evidence is scarcer (no studies on perennial AR and the only desirable outcome for which INAH+INCS are favored over INAH is rhinoconjunctivitis‐related quality‐of‐life).
- **Implementation considerations**: None specific.

##### 5.3.3.1 Brief Justification

- **Efficacy and safety**
  ○ A network meta‐analysis suggested that, compared to INAH, INAH+INCS are associated with an improvement in nasal symptoms in seasonal and perennial AR (25% and 45% probability of nontrivial improvement in nasal symptoms, respectively).
  ○ For ocular symptoms, either differences between treatments were trivial (seasonal AR) or evidence was not found (perennial AR).
  ○ INAH+INCS displayed a higher probability of resulting in a nontrivial improvement of RQLQ in patients with seasonal AR (83%) than in patients with perennial AR (16%).
  ○ Similar frequencies of adverse events were observed with INAH+INCS and INAH (trivial difference). Serious adverse events associated with these interventions are rare and most of those reported in RCTs have been judged unlikely to be related to the treatment.
- **Resources required, cost‐effectiveness and equity**: A survey of ARIA experts suggested INAH+INCS to be more expensive than INAH in 23 out of 27 countries for which data were available. However, INAH+INCS are likely cost‐effective in most countries both when considering a willingness‐to‐pay of $50,000/QALY gained or of one time the GDP per capita/QALY gained (the only exception may be some low‐income countries). No INAH+INCS or INAH are on the WHO List of Essential Medicines.
- **Acceptability and feasibility**: MASK‐air data suggest that INAH+INCS are associated with higher adherence and treatment satisfaction, as well as with lower odds of being used in co‐medication (a proxy of poor rhinitis control). In addition, INAH+INCS seem to display a faster onset of action.
- **Planetary health**: No specific evidence was found in terms of comparative impact on planetary health, even though it is possible that the production of two active compounds may have a higher environmental impact than the production of one.

#### 5.3.4 Should an Intranasal H_1_‐Antihistamine vs. an Intranasal Chromone Be Used for the Treatment of AR?

**Link for the full EtD:** https://www.mask‐air.com/etd_nasal/11/.

**Context:** INAH and intranasal chromones are two alternatives that are often used in AR patients, including those with corticosteroid‐phobia or who desire a fast onset of action.

**Recommendation:** In patients with AR, we suggest using INAH over intranasal chromones. (Conditional recommendation based on very low certainty of evidence).

- **Considerations in children and adolescents**: The recommendation is applicable to children and adolescents.
- **Implementation considerations**: None specific.

##### 5.3.4.1 Brief Justification

- **Efficacy and safety**
  ○ Existing evidence is scarce and contradictory but overall suggests that intranasal antihistamines are associated with trivial or small improvements in nasal and ocular symptoms when compared to intranasal chromones.
  ○ For RQLQ, no evidence was found.
  ○ Similar frequencies of adverse events were observed with INAH and intranasal chromones (trivial difference). Serious adverse events associated with these interventions are rare and most of those reported in RCTs have been judged unlikely to be related to the treatment.
- **Resources required, cost‐effectiveness and equity**: A survey of ARIA experts suggested INAH to be more expensive than intranasal chromones in 10 out of 16 countries for which data were available (intranasal chromones are not available in many countries). However, differences in costs tended to be small. We did not identify cost‐effectiveness studies comparing INAH to intranasal chromones. No INAH or intranasal chromone is on the WHO List of Essential Medicines.
- **Acceptability and feasibility**: MASK‐air data suggest that INAH and intranasal chromones (i) are used in co‐medication at a similar frequency, and (ii) are associated with similar adherence. However, INAH are associated with higher treatment satisfaction.
- **Planetary health**: No specific evidence was found in terms of comparative impact on planetary health.

## 6 Conclusions

Intranasal medications, in particular INCS, INAH and INAH+INCS, are part of the mainstay of AR treatment. In ARIA 2024–2025, we formulated recommendations on 11 questions concerning AR intranasal treatment. Overall, we suggest using INAH+INCS over INAH or INCS and INCS over INAH. However, decisions on AR treatment should consider the clinical variability of the disease, patients' values and preferences and the affordability of treatment options.

Questions on intranasal treatments had been previously addressed in past editions of the ARIA guidelines. Box 2 and Table 3 compare recommendations on intranasal treatments of ARIA 2024–2025 *vis‐à‐vis* ARIA 2010/2016 guidelines. In brief, four questions were addressed in ARIA 2024–2025 for the first time, including questions comparing individual INCS and individual INAH+INCS. Among the remaining questions, a change in the strength and/or the directionality of recommendations was observed in three questions. For example, differently from the ARIA 2016 guidelines, we now suggest the use of INAH+INCS over INCS (particularly among patients with more severe symptoms). These changes reflect not only a larger amount of evidence from RCTs but also the evaluation of other evidence sources, such as mHealth data and results of a survey of ARIA experts. Considering these data sources was crucial to inform on criteria such as the acceptability and resources required for the interventions, rendering the guideline more person‐centered. In addition, since these data reflect information from different countries—MASK‐air is available in 30 countries, and the survey was answered by experts from more than 40 countries—their incorporation renders ARIA 2024–2025 more easily tailored to different contexts.

BOX 2 **Summary of what is new in the ARIA 2024–2025 guidelines in comparison to ARIA 2010–2016 guidelines.**

**Table:** 

New questions Should an INAH+INCS vs. no treatment be used for the treatment of AR? ○ Recommendation: “In patients with AR in whom monotherapy is unlikely to lead to significant improvement in symptoms, we recommend using INAH+INCS over no treatment. (Strong recommendation based on moderate certainty of evidence for seasonal AR and very low certainty of evidence for perennial AR)” Should any specific INCS vs. other INCS be used for the treatment of AR? ○ Recommendation: “In patients with AR, we suggest using specific INCS (in particular, fluticasone furoate or fluticasone propionate) over others (namely, beclomethasone, budesonide, ciclesonide, mometasone and triamcinolone). (Conditional recommendation based on very low or low certainty of evidence for most comparisons)” Should any specific INAH+INCS vs. other INAH+INCS be used for the treatment of AR? ○ Recommendation: “In patients with AR, we suggest using azelastine‐fluticasone over olopatadine‐mometasone. (Conditional recommendation based on moderate certainty of evidence for seasonal AR)” Should a combination of an INCS and an intranasal decongestant vs. an INCS alone be used for the treatment of AR? ○ Recommendation: “In patients with AR, we suggest against using a combination of an INCS + intranasal decongestant over an INCS alone. (Conditional recommendation based on very low certainty of evidence)” Questions with changed recommendation (in terms of directionality or strength): Should an INAH vs. no treatment be used for the treatment of AR? ○ Recommendation changed from *conditional recommendation in favor of INAH* (2010/2016 guidelines) to a *strong recommendation in favor of INAH* (2024–2025 guidelines). Should an intranasal decongestant vs. no treatment be used for the treatment of AR? ○ Recommendation changed from *conditional recommendation in favor of intranasal decongestants* (2010/2016 guidelines) to a *conditional recommendation against the intervention* (2024–2025 guidelines). Should a combination of an INAH+INCS vs. an INCS alone be used for the treatment of AR? ○ Recommendation changed from *conditional recommendation either for either INAH + INCS or INCS* (2010/2016 guidelines) to a *conditional recommendation in favor of INAH + INCS* (2024–2025 guidelines). AR, Allergic rhinitis; ARIA, Allergic Rhinitis and its Impact on Asthma; INAH, Intranasal H1‐antihistamines; INAH+INCS, Fixed combinations of intranasal H1‐antihistamines and intranasal corticosteroids; INCS, Intranasal corticosteroids.

**TABLE 3:** Comparison of the recommendations on intranasal treatments of the ARIA 2024–2025 and of the ARIA 2010/2016 guidelines. Recommendations of the ARIA 2024–2025 guidelines are highlighted by a shade in a cell; recommendations of the ARIA 2010/2016 guidelines are highlighted by a border in a cell. Shade/border color code: Green = High certainty of evidence; Yellow = Moderate certainty of evidence; Orange = Low certainty of evidence; Red = Very low certainty of evidence.

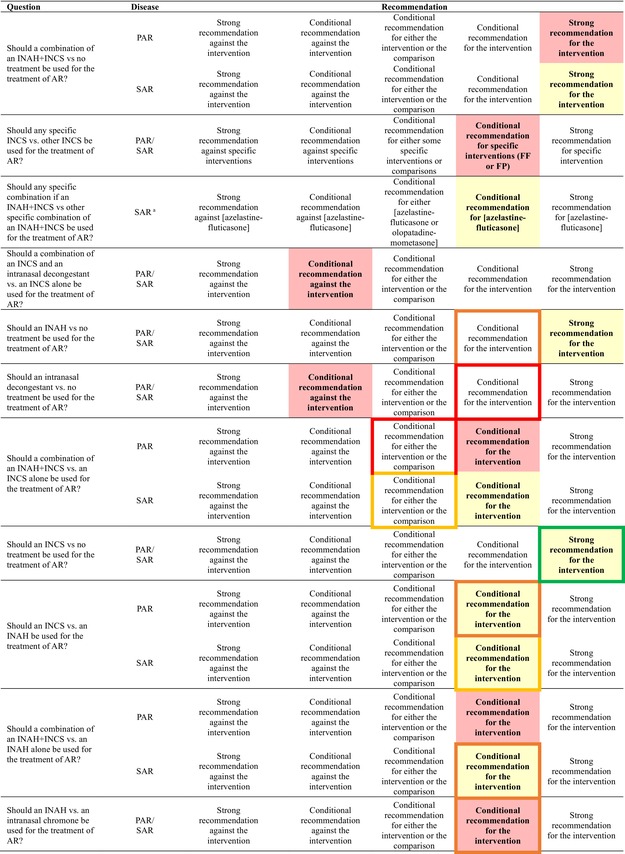

Abbreviations: AR, allergic rhinitis; FF, fluticasone furoate; FP, fluticasone propionate; INAH, intranasal antihistamines; INCS, intranasal corticosteroids; PAR, perennial allergic rhinitis; SAR, seasonal allergic rhinitis.

^a^ No evidence for PAR.

Despite the new evidence incorporated in ARIA 2024–2025, there are still some knowledge gaps that would merit further research. There is a relative lack of RCTs assessing specific subgroups of participants, including those with mild disease, those with comorbid asthma or conjunctivitis, patients from ethnic minorities, older people and—for some outcomes—children. In addition, differences in the effect of interventions by sex have not been explored. Cost‐utility studies comparing different treatments in AR are also lacking and, for some questions, we were not able to perform or include any study addressing the cost‐effectiveness criterion. Finally, there is insufficient evidence on the planetary health impact of AR interventions (with no life cycle assessment studies having been performed for such treatments), precluding this criterion from playing a decisive role in most recommendations.

Contrary to previous ARIA guidelines, we opted not to present separate recommendations for seasonal vs. perennial AR. This decision is grounded on (i) recent studies pointing to the higher relative importance of severity over disease duration [32, 33], and (ii) the fact that our SR and meta‐analyses usually found an agreement between results observed for patients with seasonal and perennial AR [23, 25]. However, since the certainty of evidence was often different between seasonal and perennial AR, there were some recommendations for which we highlighted such differences. Of note, we referred to “perennial” or “seasonal AR” considering that most RCTs did not adopt the ARIA classification (which classifies AR as “persistent” or “intermittent” [6]).

Our recommendations assume a correct use of the different intranasal medications, with inadequate use potentially resulting in lower efficacy and safety concerns [34, 35] (a video teaching patients how to use intranasal sprays can be found at https://www.youtube.com/watch?v=_ytYj1TLojM). In addition, variations in treatment duration were not explored. Future documents of the ARIA 2024–2025 guidelines will address the question of whether patients should take medications chronically or on an as‐needed basis.

These guidelines have limitations. For desirable and undesirable effects, evidence was mostly obtained from RCTs. While RCTs are the paradigm for assessing the efficacy of interventions, they tend to be associated with generalisability concerns (e.g., with overrepresentation of patients with more severe disease [36]). Also, there was a relative lack of evidence on the differential impact of AR medications in patients with and without asthma. Finally, there were several comparisons on intranasal treatments of AR that we did not assess, as the corresponding questions were not prioritized [15]. The evaluated interventions were all pharmacological in nature, but it is important to note that nonpharmacological intranasal interventions (e.g., nasal washes) are often done by patients with AR. Accordingly, the off‐label use of products for AR was not evaluated in these guidelines.

There are also important strengths associated with ARIA 2024–2025. We have followed the GRADE approach, using EtDs to develop recommendations. Additionally, we have used several approaches to formulate guideline questions and considered different data sources. Finally, we have conducted several SR and meta‐analyses to provide updated evidence on the desirable and undesirable effects of interventions. These SR have been complemented by several other evidence sources (e.g., mHealth data, pharmacovigilance data and data from a survey of experts). These sources of data have an international scope, facilitating the tailoring of the ARIA guidelines to different regions or contexts.

In conclusion, this report compared intranasal treatments for the management of AR. The recommendations were developed following the GRADE approach and consider evidence from multiple sources, including systematic reviews of randomized controlled trials, mHealth data and a survey of experts.

## Author Contributions

Bernardo Sousa‐Pinto, Jean Bousquet, Holger J. Schünemann and Torsten Zuberbier were responsible for the coordination of the project (as members of the ARIA 2024–2025 guidelines steering committee) and contributed to the methodology (including evidence synthesis and analysis), discussion of the evidence and drafting of recommendations (as members of the ARIA 2024–2025 guideline panel) and writing the manuscript. Rafael José Vieira and Antonio Bognanni contributed to the methodology (including evidence synthesis and analysis), discussion of the evidence and drafting of recommendations (as members of the ARIA 2024–2025 guideline panel) and writing the manuscript. Arunas Valiulis, Sian Williams, Anna Bedbrook, Maria Jose Torres, G Walter Canonica, Leticia de las Vecillas, Mark S. Dykewicz, Cristina Jacomelli, Ludger Klimek, Lucas Leemann, Olga Lourenço, Yuliia Palamarchuk, Nikolaos G. Papadopoulos, Ana Margarida Pereira, Marine Savouré, Sanna K. Toppila‐Salmi, Maria Teresa Ventura, Juan José Yepes‐Nuñez, Elena Azzolini, Gilles Louis, Elena Parmelli and Jaron Zuberbier contributed to the discussion of the evidence and drafting of recommendations (as members of the ARIA 2024–2025 guideline panel) and writing the manuscript. Rita Amaral, Sara Gil‐Mata, Manuel Marques‐Cruz, Ewa Borowiack, Raquel Albuquerque Costa, Olga Mariana Cunha, Renato Ferreira‐da‐Silva, Despo Ierodiakonou, Justyna Litynska, Inês Ribeiro‐Vaz, Ewelina Sadowska, Tuuli Thomander and João A. Fonseca contributed to the methodology (including evidence synthesis and analysis) and revising and editing the manuscript. All other authors were part of the international panel revising the recommendations, contributing to the guidelines by providing feedback on the recommendations and revising and editing the manuscript.

## Disclosure

Dr. Alkis Togias' co‐authorship of this report does not constitute endorsement by the National Institute of Allergy and Infectious Diseases, the National Institutes of Health or any other Agency of the United States Government.

## Conflicts of Interest

J. Bousquet reports personal fees from Cipla, Menarini, Mylan, Novartis, Purina, Sanofi‐Aventis, Teva, Noucor, other from KYomed‐Innov, other from Mask‐air‐SAS, outside the submitted work. D. Larenas Linnemann reports personal fees from ALK, Armstrong, Astrazeneca national and global, Bayer, Chiesi, Grunenthal, Grin, GSK national and global, Viatris, Menarini, MSD, Novartis, Pfizer, Sanofi, Siegfried, Carnot, Syneos Health, grants from Abbvie, Bayer, Lilly, Sanofi, Astrazeneca, Pfizer, Novartis, Pulmonair, GSK, Chiesi, Biopharma, outside the submitted work; and Editor in chief of Immune System (Karger)–member of asthma committee ACAAI–subgroup chair of allergen immunotherapy Practice parameter update JTF AAAAI/ACAAI 2024–member of allergen immunotherapy committee AAAAI–chair of allergen immunotherapy committee CMICA–member of allergic asthma task force EAACI. M. Blaiss reports personal fees from Opella, personal fees from GSK, personal fees from Sanofi, personal fees from AstraZeneca, personal fees from Bayer, during the conduct of the study. M. Worm reports other from AbbVie Deutschland GmbH & Co. KG, other from Aimmune Therapeutics UK Limited, other from ALK‐Abelló Arzneimittel GmbH, other from Allergopharma GmbH & Co KG, other from Almirall Hermal GmbH, other from Amgen GmbH, other from AstraZeneca GmbH, other from Bayer AG, other from Bencard Allergy GmbH, other from Bioprojet Pharma, other from Boehringer Ingelheim Pharma GmbH &Co.KG, other from Bristol Myers Squibb GmbH & Co. KGaA, other from Galderma Laboratorium GmbH, other from Glaxo Smith Kline GmbH & Co. KG, other from Infectopharm Arzneimittel und Consilium GmbH, other from LEO Pharma GmbH, other from Lilly Deutschland GmbH, other from Mylan Germany GmbH (A Viatris Company), other from Novartis AG, other from Octapharma AG, other from Pfizer Pharma GmbH, other from Sanofi‐Aventis Deutschland GmbH/Ðenzyme Europe B. B.Ð, outside the submitted work. A. Boner reports that Envicon Medical SRL belongs to my son Tommaso. Envicon Medical produces a nutraceutical (Auxilie Immuplus) with antioxidant activity. P. Devillier reports personal fees and nonfinancial support from Astra Zeneca, personal fees from Chiesi, personal fees from GlaxoSmithKline, personal fees from Menarini, personal fees from Viatris, personal fees from Meda Pharma, personal fees from ALK‐Abello, personal fees and nonfinancial support from Stallergenes, outside the submitted work. N. Papadopoulos reports personal fees from Nestlé Nutrition Institute, personal fees from Abbott Nutrition, grants from Numil Hellas SA, grants from VIANEX, personal fees from GSK, personal fees from HAL Allergy Holding B.V, personal fees from Menarini International Operations Luxembourg SA, personal fees from Regeneron Pharmaceuticals Inc., personal fees from Berlin—Chemie AG, personal fees from DBV Technologies SA, grants from Vibrant America, personal fees from Hyproca Nutrition USA INC, personal fees from Danone Trading Medical B.V., personal fees from Med Maps srl, outside the submitted work. R. Naclerio reports personal fees from Lyra, personal fees from Sanofi, outside the submitted work. S. Toppila‐Salmi reports grants and other support from GSK, grants and other support from Sanofi, other support from AstraZeneca, other support from ALK‐Abelló, others support from OrionPharma, outside the submitted work. J. Bernstein reports grants from Allergy Therapeutics, grants from ALK, outside the submitted work. I am also on the JTF for the AAAAI/ACAAI and co‐author on the Rhinitis guidelines. Also, Chairman of the AAAAI Foundation and a member of the WAO board of directors. B. Gradauskiene reports personal fees from Viatris, personal fees from Berlin‐Chemie Menarini, grants and personal fees from AstraZeneca, personal fees from Mylan Healthcare, personal fees from AbbVie, outside the submitted work. M. Wagenmann reports personal fees from Allergopharma, personal fees from ALK‐Abello, grants and personal fees from AstraZeneca, personal fees from CSL Behring, grants and personal fees from GSK, personal fees from HAL, personal fees from Leti Pharma, personal fees from MSD, personal fees from Novartis, grants and personal fees from Regeneron, grants and personal fees from Sanofi, personal fees from Stallergenes, grants from Takeda, outside the submitted work. I. Pali‐Schöll reports speaker fees from Bencard GmbH. M. Kupczyk reports personal fees from Adamed, personal fees from Astra Zeneca, personal fees from Abbvie, personal fees from Chiesi, personal fees from Berlin Chemie Menarini, personal fees from Glenmark, personal fees from GSK, personal fees from Novartis, personal fees from Sanofi, personal fees from Stada, personal fees from HVD, personal fees from Emma, personal fees from Celon Pharma, personal fees from Lek‐Am, personal fees from Teva, outside the submitted work. J. SASTRE reports grants and personal fees from SANOFI, personal fees from GSK, personal fees from NOVARTIS, personal fees from ASTRA ZENECA, personal fees from MUNDIPHARMA, personal fees from FAES FARMA, outside the submitted work. A. Cruz reports personal fees from AstraZeneca, personal fees from CHIESI, personal fees from GSK, personal fees from Eurofarma, personal fees from Sanofi, personal fees from Farmoquimica, personal fees from Sunvou, outside the submitted work. M. Soyka reports other from Sanofi, other from GSK, other from MSD, other from Astra Zeneca, other from Novartis, outside the submitted work. J. Mullol reports personal fees and other from SANOFI‐GENZYME and REGENERON, grants, personal fees and other from VIATRIS/MEDA Pharma, grants and personal fees from NOUCOR/URIACH Group, personal fees from Menarini, personal fees from UCB, personal fees and other from AstraZeneca, grants, personal fees and other from GSK, personal fees from MSD, personal fees and other from Lilly, personal fees and other from GLENMARK, outside the submitted work. T. Casale reports grants and others from ELI LILLY, outside the submitted work. D. Ryan reports personal fees from Thermo‐Fisher, personal fees from Menarini, personal fees from Viatris, outside the submitted work. D. Sakurai reports grants and personal fees from Torii, grants and personal fees from Tanabe Mitsubishi, personal fees from Shionogi, grants and personal fees from Taiho, grants and personal fees from Kyorin, personal fees from Meiji Seika Pharma, personal fees from Thermo fisher scientific diagnostics, grants and personal fees from Tsumura, personal fees from Hisamitsu, personal fees from Novartis, personal fees from Sanofi, grants and personal fees from Astra Zeneca, grants from Eli Lilly, outside the submitted work. C. Suppli Ulrik reports grants, personal fees and other from AstraZeneca, grants, personal fees and other from Boehringer Ingelheim, grants, personal fees and other from Sanofi Genzyme, personal fees and other from GlaxoSmithKline, personal fees from Berlin‐Chemie Menarini, personal fees from Novartis, personal fees and other from Teva, personal fees from Orion Pharma, personal fees and other from TFF Pharmaceuticals, personal fees and other from Pfizer, personal fees and other from Chiesi, personal fees from Covis Pharma, personal fees from Takeda, personal fees from Hikma Pharmaceuticals, personal fees from Novo Nordisk, personal fees from Roche, other from European Respiratory Society, outside the submitted work. T. Zuberbier reports personal fees from Amgen, personal fees from AstraZeneca, personal fees from AbbVie, personal fees from ALK‐Abelló, personal fees from Almirall, personal fees from Astellas, personal fees from Bayer Health Care, personal fees from Bencard, personal fees from Berlin Chemie, personal fees from FAES Farma, personal fees from HAL Allergie GmbH, personal fees from Henkel, personal fees from Kryolan, personal fees from Leti, personal fees from L'Oreal, personal fees from Meda, personal fees from Menarini, personal fees from Merck Sharp & Dohme, personal fees from Novartis, personal fees from Nuocor, personal fees from Pfizer, personal fees from Sanofi, personal fees from Stallergenes, personal fees from Takeda, personal fees from Teva, personal fees from UCB, personal fees from Uriach, personal fees from Abivax, personal fees from Blueprint, personal fees from Celldex, personal fees from Celltrion, outside the submitted work; and Committee member, “Allergic Rhinitis and its Impact on Asthma” (ARIA), Member of the Board, German Society for Allergy and Clinical Immunology (DGAKI), Head, European Centre for Allergy Research Foundation (ECARF), President, Global Allergy and Asthma Excellence Network (GA^2^LEN), and Member, Committee on Allergy Diagnosis and Molecular Allergology, World Allergy Organization (WAO). M. Recto reports personal fees from VIATRIS, personal fees from GLENMARK, outside the submitted work. S. Williams reports grants from ALK Abello, outside the submitted work. E. Compalati reports personal fees from Lofarma spa, during the conduct of the study. J. Correia‐de‐Sousa reports other from Boheringer Ingelheim, personal fees and other from GSK, grants, personal fees and other from AstraZeneca, nonfinancial support and other from Bial, from Mundipharma, personal fees and other from Sanofi, from Novartis, personal fees from MSD, personal fees from Medinfar, outside the submitted work. L. Cecchi reports personal fees from Astra Zeneca, personal fees from GSK, personal fees from Novartis, personal fees from Chiesi, personal fees from Sanofi, personal fees from Menarini, personal fees from Thermofisher, personal fees from Firma, outside the submitted work. V. Cardona reports personal fees from Allergy Therapeutics, personal fees from Probelte, personal fees from Roxall, other from Thermofisher, personal fees from Viatris, personal fees from GSK, outside the submitted work. J. Davies reports grants from the Australian National Health and Medical Research Council, grants from the Australian Research Council, grants from the Australian Research Council, grants from NHMRC Clinical Trials and Cohort Studies 2023, grants from MRFF Chronic Respiratory Conditions, grants from Bayer Healthcare LLC USA, nonfinancial support from Swisens SA Switzerland, nonfinancial support from Kenelec Australia, outside the submitted work; in addition, Dr. Davies' organization QUT has a patent US PTO 14/311944 issued. P. Keith reports personal fees from ALK, personal fees from Bausch, personal fees from the Canadian Agency for Drugs and Technologies in Health, personal fees from Bayer, personal fees from GSK, personal fees from Medexus, personal fees from Novartis, personal fees from Sanofi, outside the submitted work. J. Litynska reports from the Fraunhofer Institute, during the conduct of the study. M. Makris reports personal fees from MENARINI, personal fees from ASTRA ZENECA, personal fees from GSK, personal fees from CHIESI, personal fees from Novartis, personal fees from ELPEN, outside the submitted work. G. Paoletti reports fees for speaker activities and/or advisory board participation from Lofarma, GSK, and AstraZeneca, outside the submitted work. I. Tsiligianni reports grants from Chiesi, GSK Hellas, Menarini, Astra Zeneca Greece, outside the submitted work. T. Haahtela reports personal fees from Orion Pharma, personal fees from ALK Nordic, outside the submitted work. H. Kraxner reports Speaker's fee and congress support from Sanofi—Speaker's fee and congress support from Viatris—Speaker's fee and congress support from Berlin‐Chemie—Speaker's fee and congress support from Ewopharma—Advisory Board membership: Sanofi—Advisory Board membership: Berlin‐Chemie. M. Hyland reports a grant from GSK, grants from AstraZeneca, from null, outside the submitted work. I. Ansotegui reports personal fees from Bayer, personal fees from Eurodrug, personal fees from Gebro, personal fees from Menarini, personal fees from MSD, personal fees from Roxall, personal fees from Sanofi, personal fees from Cipla, personal fees from Glenmark, personal fees from Opella, personal fees from GSK, outside the submitted work. P. Werminghaus reports personal fees from Astrazeneca, personal fees from Bencard Allergy, personal fees from Glaxosmithkline, personal fees from Sanofi, personal fees from Stallergenes, outside the submitted work. H. Olze reports personal fees from F. Hoffmann‐La Roche Ltd., Sanofi‐Aventis Deutschland GmbH, AstraZeneca GmbH, GlaxoSmithKline GmbH and Co., KG, Novartis, outside the submitted work. A. Todo‐Bom reports personal fees from GSK, personal fees from AstraZeneca, personal fees from Leti, personal fees from Almirall, grants from Abbvie, outside the submitted work. O. Palomares reports research grants from MINECO, Ministerio de Ciencia e Innovación, CAM, Inmunotek S.L., Novartis, and AstraZeneca and fees for giving scientific lectures or participation in Advisory Boards from AstraZeneca, Pfizer, GlaxoSmithKline, Inmunotek S.L., Novartis, Regeneron and Sanofi. R. Buhl reports personal fees from AstraZeneca, Berlin‐Chemie, Celltrion, Chiesi, Cipla, Sanofi, and Teva, as well as grants to Mainz University Hospital and personal fees from Boehringer Ingelheim, GlaxoSmithKline, Novartis, and Roche, outside the submitted work. Y. Okamoto reports personal fees from Torii Pharmaceutical Co. Ltd, personal fees from Tanabe‐Mitsubishi Pharmaceutical Co. Ltd., personal fees from Kirin Holdings Co. Ltd., personal fees from Shionogi Co. Ltd., personal fees from Stallergenes‐Greer, personal fees from Diichi‐Sankyo, outside the submitted work. M. Giovannini reports personal fees from Sanofi, Thermo Fisher Scientific, outside the submitted work. N. Roche reports grants and personal fees from GSK, personal fees from AstraZeneca, grants and personal fees from Chiesi, grants and personal fees from Pfizer, personal fees from Sanofi, personal fees from Zambon, personal fees from MSD, personal fees from Austral, personal fees from Biosency, outside the submitted work. L. Taborda‐Barata reports personal fees from Sanofi, personal fees from LETI, outside the submitted work. P. Kuna reports personal fees from Adamed, personal fees from Angellini, personal fees and nonfinancial support from AstraZeneca, personal fees from Berlin Chemie Menarini, personal fees from Celon Pharma, personal fees from Chiesi, personal fees from Glenmark, personal fees from GSK, personal fees from Polpharma, personal fees from Sanofi, personal fees from Teva, personal fees from Zentiva, outside the submitted work. F. Serpa reports personal fees from Astra Zeneca, personal fees from Sanofi, personal fees from Takeda, personal fees from CSL, personal fees from Novartis, outside the submitted work.M. Ollert reports personal fees from Allergy Therapeutics, personal fees from Thermo Fisher, personal fees from Hycor Diagnostics, outside the submitted work. O. Pfaar reports grants and personal fees from ALK‐Abelló, grants and personal fees from Allergopharma, grants and personal fees from Stallergenes Greer, grants and personal fees from HAL Allergy Holding B.V./HAL Allergie GmbH, grants and personal fees from Bencard Allergie GmbH/Allergy Therapeutics, grants and personal fees from Laboratorios LETI/LETI Pharma, grants and personal fees from GlaxoSmithKline, personal fees from ROXALL Medizin, personal fees from Novartis, grants and personal fees from Sanofi‐Aventis and Sanofi‐Genzyme, personal fees from Med Update Europe GmbH, personal fees from streamedup! GmbH, personal fees from Pohl‐Boskamp, grants from Inmunotek S.L., personal fees from John Wiley and Sons, AS, personal fees from Paul‐Martini‐Stiftung (PMS), personal fees from Regeneron Pharmaceuticals Inc., personal fees from RG Aerztefortbildung, personal fees from Institut für Disease Management, personal fees from Springer GmbH, grants and personal fees from AstraZeneca, personal fees from IQVIA Commercial, personal fees from Ingress Health, personal fees from Wort&Bild Verlag, personal fees from Verlag ME, personal fees from Procter&Gamble, personal fees from ALTAMIRA, personal fees from Meinhardt Congress GmbH, personal fees from Deutsche Forschungsgemeinschaft, personal fees from Thieme, grants from Deutsche AllergieLiga e.V., personal fees from AeDA, personal fees from Alfried‐Krupp Krankenhaus, personal fees from Red Maple Trials Inc., personal fees from Königlich Dänisches Generalkonsulat, personal fees from Medizinische Hochschule Hannover, personal fees from ECM Expro&Conference Management, personal fees from Technical University Dresden, grants and personal fees from Lilly, personal fees from Japanese Society of Allergy, personal fees from Forum für Medizinische Fortbildung, personal fees from Dustri‐Verlag, personal fees from Pneumolive, grants and personal fees from ASIT Biotech, personal fees from LOFARMA, personal fees from Paul‐Ehrlich‐Institut, personal fees from Almirall, outside the submitted work; and Vice President and member of EAACI Excom, member of ext. board of directors DGAKI; coordinator, main‐ or co‐author of different position papers and guidelines in rhinology, allergology and allergen‐immunotherapy; Editor‐in‐Chief (EIC) of Clinical Translational Allergy (CTA), Associate Editor (AE) of Allergy. M. Moniuszko reports having received in the past personal fees and other from Berlin‐Chemie/Menarini, personal fees and other from Astra Zeneca, personal fees and other from GlaxoSmithKline, personal fees and other from Novartis, personal fees and other from Chiesi, personal fees and other from Celon Pharma, personal fees and other from Takeda, personal fees and other from Polfarmex, personal fees and other from CSL Behring, personal fees from Glenmark Pharmaceuticals, personal fees and other from Sanofi, personal fees and other from Teva, outside the submitted work. T. Thomander has received personal research grants from the Finnish ORL‐HNS Foundation, the Research Foundation of the Pulmonary Diseases, the Maud Kuistila Memorial Foundation, and the Foundation of the Finnish Anti‐Tuberculosis Association, supporting the work on this study. The funding bodies had no role in study design, data collection, analysis, interpretation, or manuscript preparation. S. Del Giacco reports grants and personal fees from AstraZeneca, grants and personal fees from GSK, grants and personal fees from Sanofi, personal fees from Chiesi, and personal fees from Menarini, outside the submitted work. M. Bourgoin‐Heck reports nonfinancial support from Thermofisher, personal fees from ALK, personal fees from Takeda, personal fees from Biocryst, personal fees from DBV, outside the submitted work. H. Schünemann reports developed guidelines on Allergic Rhinitis in Asthma (ARIA) and his academic institution received research funding for it. The other authors have nothing to declare outside the submitted work.

## Supporting information


**Appendix S1:** all70131‐sup‐0001‐AppendixS1.DOC.

## Data Availability

The data that support the findings of this study are available from the corresponding author upon reasonable request.
