# Supplementary material for: Allergic Rhinitis and Its Impact on Asthma (ARIA)‐EAACI Guidelines—2024–2025 Revision: Part I—Guidelines on Intranasal Treatments
Source: Allergy. 2025 Dec 1;81(4):954–76. doi: 10.1111/all.70131 (PMC13040648; doi:10.1111/all.70131)
Supplement: Supplementary file 1 — Appendix S1: all70131‐sup‐0001‐AppendixS1.DOC. [file ALL-81-954-s001.doc]

**Online supplement of the Allergic Rhinitis and its Impact on Asthma (ARIA) guidelines – 2024/2025 revision: Part I – Guidelines on intranasal treatments**

**Brief justification of question 1: Should a combination of an intranasal H1-antihistamine and an intranasal corticosteroid vs. no treatment be used for the treatment of allergic rhinitis?**

- Efficacy and safety:
  - Results from a network meta-analysis suggested that, compared to placebo, INAH+INCS are associated with a 100% probability of resulting in a non-trivial improvement in nasal symptoms in seasonal AR. In perennial AR, this probability was of 89%.
  - INAH+INCS displayed a 100% probability of resulting in a non-trivial improvement in ocular symptoms in patients with seasonal AR. For perennial AR, no evidence was found.
  - INAH+INCS displayed a high probability of resulting in a non-trivial improvement of rhinoconjunctivitis-related quality-of-life in patients with seasonal AR (100%) and in patients with perennial AR (67%).
  - INAH+INCS were associated with a trivial increase in the risk of adverse events compared to placebo. Serious adverse events associated with INAH+INCS are rare and most of those reported in RCTs have been judged unlikely to be related to the treatment.
- Resources required, cost-effectiveness and equity: A survey to ARIA experts suggested the costs of INAH+INCS to vary widely across countries. However, their weekly costs appear to be lower than the costs resulting from productivity losses in patients with poorly controlled AR. Considering only direct medical costs, INAH+INCS were found to be cost-effective when considering a willingness-to-pay of $50,000/QALY gained or of one time the GDP per capita/QALY gained (the only exception with the latter thresholds may be some low- or middle-income countries). No INAH+INCS is on the WHO List of Essential Medicines.
- Acceptability and feasibility: MASK-air® data suggest that INAH+INCS are associated with high treatment satisfaction and with lower odds of being used in co-medication than other commonly used drug classes (e.g., OAH or INAH). Medication adherence in the pollen season appears to be similar to the one observed for OAH and INCS. Moreover, INAH display a fast onset of action.
- Planetary health: No specific evidence was found in terms of the impact of INAH+INCS on planetary health. The impact for production, packaging and transport of INAH+INCS may be offset by the reduced emissions that a better level of AR control may ensue (i.e., reduced need for healthcare visits and over-the-counter medications).

**Brief justification of question 5: Should an intranasal H1-antihistamine vs. no treatment be used for the treatment of allergic rhinitis?**

- Efficacy and safety:
  - Results from a network meta-analysis suggested that, compared to placebo, INAH are associated with a 100% probability of resulting in a non-trivial improvement in nasal symptoms in seasonal AR. In perennial AR, this probability was of 44%.
  - INAH displayed a 81% probability of resulting in a non-trivial improvement in ocular symptoms in patients with seasonal AR. For perennial AR, no evidence was found.
  - INAH displayed a high probability of resulting in a non-trivial improvement of rhinoconjunctivitis-related quality-of-life in patients with seasonal AR (94%) and in patients with perennial AR (59%).
  - INAH were associated with a trivial increase in the risk of adverse events compared to placebo. Serious adverse events associated with INAH are rare and most of those reported in RCTs have been judged unlikely to be related to the treatment.
- Resources required, cost-effectiveness and equity: A survey to ARIA experts suggested the costs of INAH to vary widely across countries. However, their weekly costs appear to be lower than the costs resulting from productivity losses in patients with poorly controlled AR. Considering only direct medical costs, INAH were found to be cost-effective when considering a willingness-to-pay of $50,000/QALY gained or of one time the GDP per capita/QALY gained (the only exception with the latter thresholds may be some low- or middle-income countries). INAH are not available in several countries and no INAH is on the WHO List of Essential Medicines.
- Acceptability and feasibility: MASK-air® data suggest that INAH are associated with moderate treatment satisfaction and with higher odds of being used in co-medication than other commonly used drug classes (e.g., OAH or INCS). Medication adherence in the pollen season appears to be lower to the one observed for OAH and INCS. However, INAH display a fast onset of action.

Planetary health: No specific evidence was found in terms of the impact of INAH on planetary health. The impact for production, packaging and transport of INAH may be offset by the reduced emissions that a better level of AR control may ensue (i.e., reduced need for healthcare visits and over-the-counter medications).

**Brief justification of question 6: Should an intranasal decongestant vs. no treatment be used for the treatment of allergic rhinitis?**

- Efficacy and safety:
  - Existing evidence is scarce and contradictory but overall suggests that intranasal decongestants are associated with a trivial impact in nasal symptoms and rhinoconjunctivitis-related quality of life in seasonal and perennial AR. Overall, the literature points to a limited role of intranasal decongestants, particularly in the short-term relief of nasal congestion.
  - For ocular symptoms, no evidence was found on the impact of intranasal decongestants.
  - Intranasal decongestants were associated with a small increase in the risk of adverse events compared to placebo. The long-term use of nasal decongestants has been linked to rhinitis medicamentosa. In additions, there have been reports of rare teratogenic effects associated with the use of intranasal decongestants, as well as of some rare serious adverse events in the elderly. Pharmacovigilance data also suggested that intranasal decongestants can be associated with dependence on the use of these drugs.
- Resources required, cost-effectiveness and equity: A survey to ARIA experts suggested that, even though there may be some across-country variability, intranasal decongestants may incur in moderate costs. No cost-effectiveness studies are available. One intranasal decongestant – xylometazoline – is on the WHO List of Essential Medicines.
- Acceptability and feasibility: In terms of acceptability, MASK-air® data suggest that intranasal decongestants are associated with moderate treatment satisfaction and with higher frequency of being used in co-medication than other commonly used drug classes (e.g., OAH or INCS). Medication adherence in the pollen season appears to be lower to the one observed for OAH and INCS. However, intranasal decongestants display a fast onset of action.
- Planetary health: No specific evidence was found in terms of the impact of intranasal decongestants on planetary health. The impact for production, packaging and transport is probably not offset by the impact of intranasal decongestants in terms of desirable effects.

**Brief justification of question 8: Should an intranasal glucocorticosteroid vs. no treatment be used for the treatment of allergic rhinitis?**

- Efficacy and safety:
  - Results from a network meta-analysis suggested that, compared to placebo, intranasal corticosteroids (INCS) are associated with a 100% probability of resulting in a non-trivial improvement in nasal symptoms in seasonal and perennial allergic rhinitis (AR).
  - For ocular symptoms, INCS displayed a 96% probability of resulting in a non-trivial improvement in the total ocular symptom score in patients with seasonal AR. In patients with perennial AR, this probability was of 44%.
  - INCS displayed a high probability of resulting in a non-trivial improvement of rhinoconjunctivitis-related quality-of-life in patients with seasonal AR (100%) and in patients with perennial AR (91%).
  - INCS were associated with a trivial increase in the risk of adverse events compared to placebo. Serious adverse events associated with INCS are rare and only one was judged to be related to the treatment (increased intra-ocular pressure). Although rare, pharmacovigilance data also suggested that INCS can be associated with glaucoma.
- Resources required, cost-effectiveness and equity: A survey to Allergic Rhinitis and its Impact on Asthma (ARIA) experts suggested the costs of INCS to vary widely internationally. However, their weekly costs appear to be lower than the costs resulting from productivity losses in patients with poorly controlled AR. Considering only direct medical costs, INCS were found to be cost-effective when considering a willingness-to-pay of $50,000/QALY gained or of one time the GDP per capita/QALY gained (the only exception with the latter thresholds may be some low-income countries). One INCS – budesonide – is on the WHO List of Essential Medicines.
- Acceptability and feasibility: MASK-air® data suggest that INCS are associated with high treatment satisfaction and with lower odds of being used in co-medication than other commonly used drug classes (e.g., oral antihistamines [OAH] or intranasal antihistamines [INAH]). Medication adherence in the pollen season appears to be similar to the one observed for OAH and INAH+INCS However, INAH display a relatively slow onset of action.
- Planetary health: No specific evidence was found in terms of the impact of INCS on planetary health. The impact for production, packaging and transport of INCS may be offset by the reduced emissions that a better level of AR control may ensue (i.e., reduced need for healthcare visits and over-the-counter medications).
